# Active PLK1-driven metastasis is amplified by TGF-β signaling that forms a positive feedback loop in non-small cell lung cancer

**DOI:** 10.1038/s41388-019-1023-z

**Published:** 2019-09-23

**Authors:** Sol-Bi Shin, Hay-Ran Jang, Rong Xu, Jae-Yeon Won, Hyungshin Yim

**Affiliations:** Department of Pharmacy, College of Pharmacy, Institute of Pharmaceutical Science and Technology, Hanyang University, Ansan, Gyeonggi-do Korea

**Keywords:** Oncogenes, Prognostic markers

## Abstract

Early findings that PLK1 is highly expressed in cancer have driven an exploration of its functions in metastasis. However, whether PLK1 induces metastasis in vivo and its underlying mechanisms in NSCLC have not yet been determined. Here, we show that the expression of active PLK1 phosphorylated at T210, abundant in TGF-β-treated lung cells, potently induced metastasis in a tail-vein injection model. Active PLK1 with intact polo-box and ATP-binding domains accelerated cell motility and invasiveness by triggering EMT reprogramming, whereas a phosphomimetic version of p-S137-PLK1 did not, indicating that the phosphorylation status of PLK1 may determine the cell traits. Active PLK1-driven invasiveness upregulated TGF-β signaling and TSG6 encoded by *TNFAIP6*. Loss of *TNFAIP6* disturbed the metastatic activity induced by active PLK1 or TGF-β. Clinical relevance shows that *PLK1* and *TNFAIP6* are strong predictors of poor survival rates in metastatic NSCLC patients. Therefore, we suggest that active PLK1 promotes metastasis by upregulating TGF-β signaling, which amplifies its metastatic properties by forming a positive feedback loop and that the PLK1/TGF-β-driven metastasis is effectively blocked by targeting PLK1 and TSG6, providing PLK1 and TSG6 as negative markers for prognostics and therapeutic targets in metastatic NSCLC.

## Introduction

Approximately 40% of the patients newly diagnosed with lung cancer, a leading cause of cancer-related mortality worldwide [1, 2], already have metastases to bone or other sites in the lung, brain, liver, and adrenal glands [3]. The 5-year survival rates of patients with non-small cell lung cancer (NSCLC), ~85% of all lung cancers, are 10–20% or less, with a median survival of 8 to 10 months [4]. Understanding and regulating the biology of metastasis is needed to improve the cancer therapies and increase the survival rates of NSCLC patients. As metastasis starts with an invasion of the tissue surrounding the primary tumor [5], it is therapeutically important to block the invasion of cancer cells through the surrounding extracellular matrix, which could serve as an intrinsic barrier to the invasion of metastatic cancer cells [6]. Cancer cells acquire the ability to migrate and invade by altering their gene expression during the epithelial-to-mesenchymal transition (EMT); thus, the EMT has a functional effect on metastasis.

The EMT can be driven by a variety of growth factors, such as transforming growth factor-β (TGF-β), acting through receptor tyrosine kinases [7]. In the tumor microenvironment, TGF-β signaling affects tumor progression by phosphorylating or interacting with numerous factors, including Smad2/3/4, Ras/MAPK/ERK, and PI3K/AKT signaling, depending on the cell context [8–10]. The activation of TGF-β signaling induces the expression of genes related to mesenchymal regulation, including *SNAI1, SNAI2, TWIST*, and *ZEB1* [8]. Although mesenchymal traits are important in migrating from the primary region, the epithelial traits remain important to proliferation and colonization in the second region. Therefore, a mesenchymal-to-epithelial transition (MET) is a possible process at the second site. However, many studies have reported the existence of a partial EMT status or mixture of mesenchymal and epithelial cells used for efficient metastasis and colonization [7, 11, 12]. The mechanisms by which those opposite properties regulate migration for metastasis and tumor progression for colonization are not fully understood.

High expression of PLK1, a proto-oncogene and critical regulator of several cellular events, including cell division, DNA replication, and DNA damage recovery [13–15], has been found in several cancers. Therefore, PLK1 has been explored its possible functions in inducing the EMT of carcinoma in the breast [16], colon [17], bladder [18], stomach [19], and prostate [20]. In gastric cancer, Cai et al. [19] showed the involvement of AKT signaling in PLK1-induced EMT. Although they reported that PLK1 overexpression in prostate cancer triggers the EMT by activating CRAF/ERK signaling, it remains unclear how PLK1 induces the EMT and which factors are the main causes of PLK1-induced EMT. Also, it has not yet been determined whether PLK1 expression by itself can induce metastasis in vivo or whether the catalytic active form of PLK1 is needed.

Structurally, PLK1 is composed of an N-terminal catalytic ATP-binding domain and a C-terminal non-catalytic domain called the *polo-box domain* (PBD), a binding region with a phosphopeptide substrate [21–24]. The activation of PLK1 is mediated by phosphorylation at T210 and S137, although the two sites function differently [22, 23, 25]. Phosphorylation at T210 of PLK1 is mainly observed in mitosis, whereas phosphorylation at S137 functions in the S phase [22, 23]. It was reported that a phosphomimetic mutant of S137 increased the catalytic activity of PLK1 or modulated substrate specificity [22–25]. The activity and cellular functions of PLK1 are closely related to its functional domains. It is not been explored whether its functional domains have specific roles for the EMT. Based on this notion, we established a systematic inducible lentiviral expression system for several versions of PLK1.

In that way, we found that catalytically active PLK1 phosphorylated at T210 was abundant in TGF-β-treated lung cells, and its expression potently induced metastasis in a tail-vein injection in vivo mouse model. In addition, the expression of different phosphomimetic mutants of PLK1 showed different phenotypes in epithelial and mesenchymal characters. Furthermore, invasive cells expressing active PLK1 phosphorylated at T210 upregulated many genes related to TGF-β signaling, which triggered metastatic properties in a positive amplification loop.

## Results

### PLK1 overexpression is correlated with a low survival rate in metastatic NSCLC patients

**Fig. 1:**
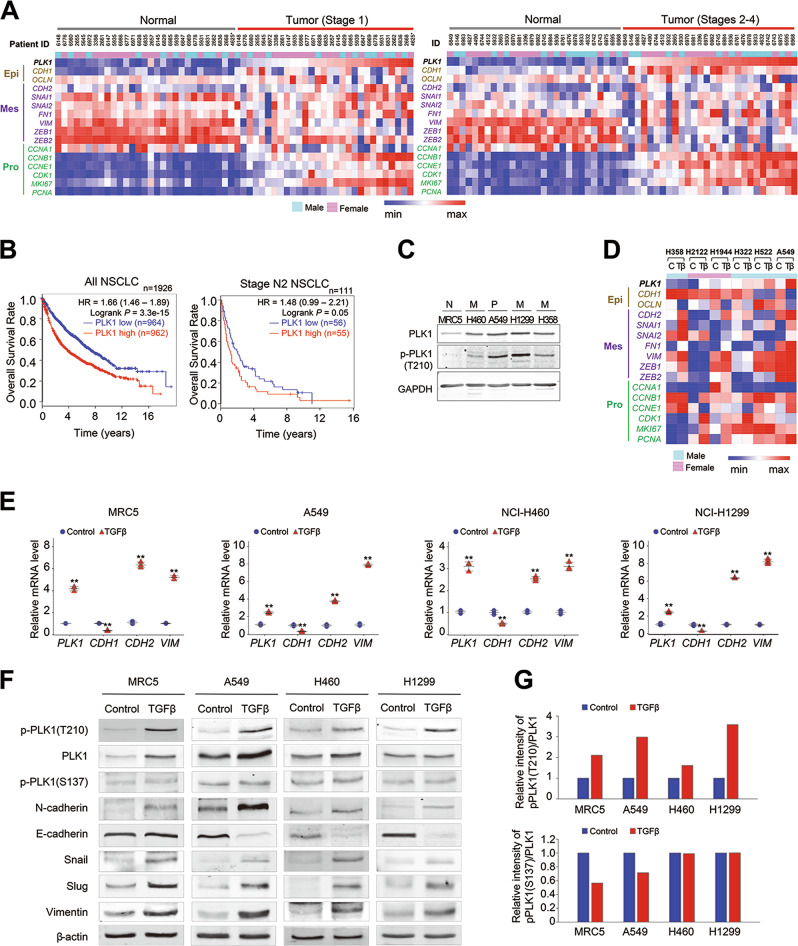
T210 of PLK1 is highly phosphorylated, but S137 is not in TGF-β-induced epithelial mesenchymal transition (EMT). **a** Heat maps were generated from TCGA lung adenocarcinoma patients’ dataset. Heat map showed the expression profile of genes, including PLK1, epithelial markers (Epi), mesenchymal markers (Mes), and proliferation markers (Pro) in paired normal and tumor tissues with stage 1 (right) or stages 2–4 (left). *4625 has missense mutation (S335C). **b** Cumulative overall survival of all NSCLC patients (left) and TMN stage N2 patients (right) according to PLK1 expression. Kaplan–Meier plot of overall survival rates for NSCLC patients was generated by splitting patients by their PLK1 expression levels (defined using median values), and the data were analyzed based on the log-rank test (left, *n* = 1926, HR = 1.66, log rank *p* = 3.3e-15; right, *n* = 111, HR = 1.48, log rank *p* = 0.05). **c** Expression and phosphorylation of PLK1 was measured using PLK1 and p-PLK1 (T210) antibodies in normal MRC5 lung cells, primary NSCLC A549, and metastatic NSCLC NCI-H460, NCI-H1299, and NCI-H358 cells. **d** A heat map analysis was performed for *PLK1*, epithelial marker *CDH1* or *OCLN*, and several mesenchymal markers, including *CDH2*, using a published transcriptome of TGF-β-treated NSCLC (GSE 114761). **e**–**g** MRC5, A549, NCI-H460, and NCI-H1299 cells were treated with 2.5 ng/ml of TGF-β for 48 h. **e** qRT-PCR was performed for *PLK1, CDH1, CDH2*, and *VIM* expression using TGF-β-treated MRC5, A549, NCI-H460, and NCI-H1299 cells. *p < 0.05; **p < 0.01; ***p < 0.001. (*n* = 3). Data presented as mean ± SD. **f** Immunoblot analyses were performed for PLK1, p-T210-PLK1, p-S137-PLK1, N-cadherin, E-cadherin, vimentin, snail, slug, and β-actin using specific antibodies with lysates from MRC5, A549, NCI-H460, and NCI-H1299 cells treated with TGF-β. **g** The band intensity values of p-T210-PLK1, p-S137-PLK1, and PLK1 were quantified using LI-COR Odyssey software (Li-COR Biosciences). The band intensity values of p-T210-PLK1 and p-S137-PLK1 were normalized to those of PLK1 and plotted

Although recent studies reported that overexpression of PLK1 induces the EMT and promotes cell motility in prostate and gastric cancer [19, 20], it is not yet well understood whether the expression of PLK1 is required to induce metastasis in vivo and in NSCLC cancer patients or which is more important in inducing the EMT for metastasis in NSCLC, the expression of PLK1 or its activation. To solve the questions, The Cancer Genome Atlas (TCGA) data was used. In the genomic analysis of patients with lung adenocarcinoma (Fig. 1a) and squamous cell carcinoma (Supplementary Fig. S1), major types of NSCLC, *PLK1* was highly expressed concomitantly with proliferation markers *MKI67* and *CCNB1*. Mesenchymal markers such as *SNAI1/2* and *FN1* were also upregulated, partially similar with *PLK1* expression in tumors of NSCLC patients (Fig. 1a and Supplementary Fig. S1). In addition, the expression levels and frequencies of PLK1 were higher in stages 2–4 than those of stage 1 of adenocarcinoma and squamous cell carcinoma (Fig. 1a and Supplementary Fig. S1), indicating that PLK1 is highly expressed in metastatic NSCLC. Then, we analyzed the clinical relevance of PLK1 expression to the cumulative overall survival (OS) rates of metastatic patients with NSCLC. Our survival analysis of 1926 NSCLC patients [26] revealed that the OS rate of NSCLC patients with high expression of PLK1 (median survival 45.8 months) was lower than that of NSCLC patients with low PLK1 expression (median survival 89 months) with high significance (*n* = 1926, HR = 1.66, log rank *p* = 3.3e-15) (Fig. 1b, left panel). We also used data from NSCLC patients with metastasis to an ipsilateral, mediastinal, or subcarinal lymph node (AJCC stage N2). The OS rate of 111 metastatic NSCLC patients with AJCC stage N2 was lower than that of all NSCLC patients (Fig. 1b). In addition, metastatic patients with high levels of PLK1 (median survival 14.9 months) had lower OS rates than metastatic patients with low PLK1 expression (median survival 25 months) (*n* = 111, HR = 1.48, log rank *p* = 0.05) (Fig. 1b, right panel). These data suggest that PLK1 overexpression is clinically correlated with low OS rates in metastatic NSCLC patients.

### T210 of PLK1 is highly phosphorylated in TGF-β-induced EMT, but S137 of PLK1 is not

To evaluate the expression and phosphorylation levels of PLK1 in normal lung cells and primary and metastatic NSCLC, we observed the protein levels of PLK1 and its active form (phosphorylated at T210) by immunoblotting in normal lung fibroblast MRC5 cells, primary NSCLC A549 cells, and metastatic NSCLC (NCI-H460, NCI-H1299, and NCI-H358) cells (Fig. 1c). As expected, in normal fibroblast MRC5 cells, the protein levels of PLK1 and p-PLK1 were lower than in NSCLC cells (Fig. 1c). Although the PLK1 and p-PLK1 levels were higher in all the NSCLC cells tested compared with normal fibroblasts, no differences in expression or phosphorylation were observed between primary and metastatic NSCLC (Fig. 1c).

To understand the expressions of PLK1 during NSCLC metastasis, we leveraged a published transcriptome of several NSCLC cells treated with TGF-β to induce the EMT (GSE 114761) [27] (Fig. 1d). For this analysis, non-EMT cell lines were ruled out based on the morphology and the changes of EMT markers, even though EMT was triggered by treatment with TGF-β. Our heat map analysis revealed that the expression of *PLK1* messenger RNA (mRNA) was increased in TGF-β-treated NSCLC cells such as A549 cells, when the mesenchymal markers, N-cadherin (*CDH2*), snail (*SNAI1*), and slug (*SNAI2*), were high and the epithelial marker E-cadherin (*CDH1*) or occludin (*OCLN*) was low. Thus, *PLK1* was upregulated during TGF-β-induced EMT in the majority of NSCLC cells analyzed, except in NCI-H358 cells (Fig. 1d).

To confirm the levels of PLK1 in TGF-β-induced EMT, we treated normal lung fibroblast MRC5 cells, primary NSCLC A549 cells, and metastatic NSCLC (NCI-H460 and NCI-H1299 cells) with 2.5 ng/ml of TGF-β. As expected, a quantitative real-time polymerase chain reaction (qRT)-PCR analysis showed that TGF-β treatment reduced the mRNA levels of the epithelial marker *CDH1* in NSCLC, whereas it increased the levels of the mesenchymal markers *CDH2* and *VIM* in the tested lung cells. The mRNA levels of *PLK1* increased 3- to 4-fold in the TGF-β-treated MRC5, A549, NCI-H460, and NCI-H1299 cells (Fig. 1e). The changes of *CDH1*, *CDH2*, and *PLK1* in the TGF-β-treated NSCLC cells were consistent with the alterations in the published transcriptome of TGF-β-treated cells (GSE 114761) (Fig. 1d, e).

Next, we examined whether the phosphorylation status of PLK1 changes during the EMT, and if so, which site is important for the EMT process. We observed the expression and phosphorylation levels of PLK1 by immunoblot analysis, using anti-phospho-S137-PLK1, anti-phospho-T210-PLK1, and anti-PLK1 antibodies in TGF-β-treated lung cells (Fig. 1f). The intensity of p-PLK1 (T210) relative to PLK1 in TGF-β–treated cells was 1.5- to 4-fold higher than in the control (Fig. 1g, upper panel). On the other hand, the intensity of p-S137-PLK1 relative to PLK1 was lower or not much altered in the TGF-β–treated cells compared with the control (Fig. 1g, lower panel). The levels of p-PLK1 at T210 changed markedly compared with those of PLK1 or p-S137-PLK1 in all the TGF-β–treated cells tested (Fig. 1f, g). Consistent with the mRNA alteration, treatment with TGF-β increased the protein levels of the mesenchymal proteins N-cadherin, vimentin, snail, and slug, whereas the protein levels of the epithelial marker E-cadherin decreased in the TGF-β-treated NSCLC but not in the normal MRC5 fibroblast cells. These data suggest that PLK1 is highly phosphorylated at T210 (but not at S137) during TGF-β–induced EMT.

### The EMT is triggered by the expression of a catalytically active mimic form of p-T210-PLK1 but not by the expression of the mimic form of p-S137-PLK1

**Fig. 2:**
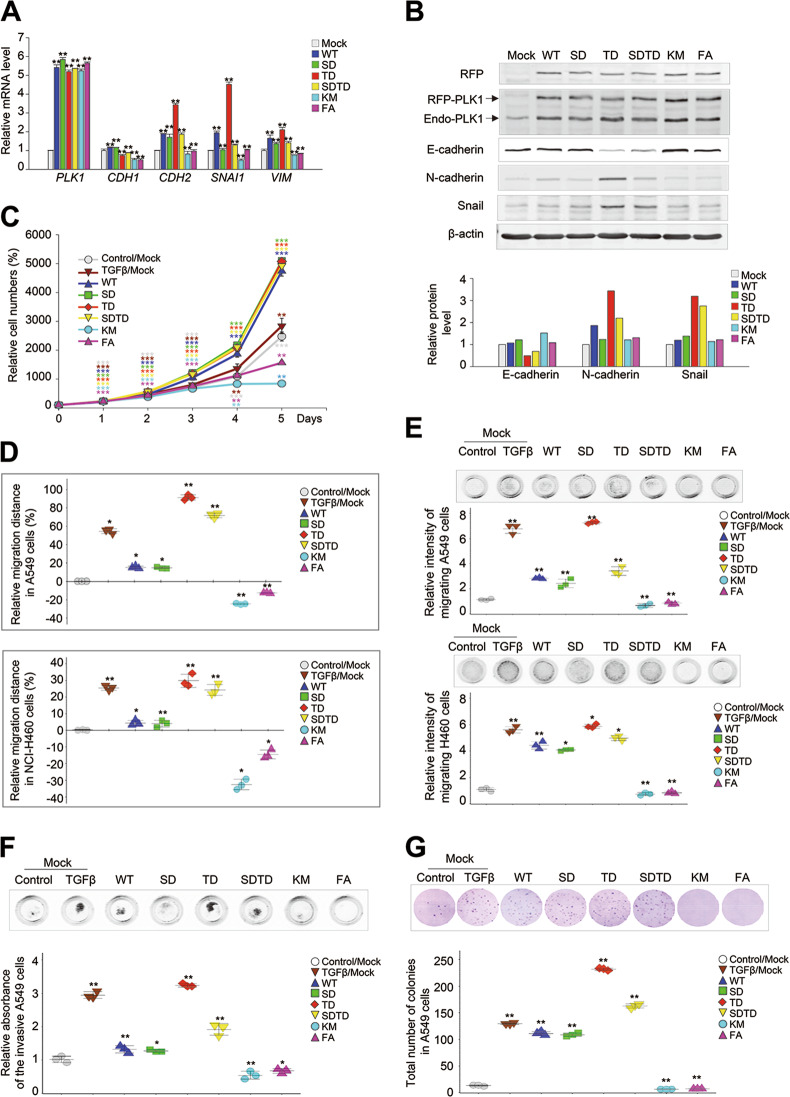
Expression of a catalytically active p-T210-PLK1 mutant induces tumorigenic and metastatic properties, including migration and invasiveness. Enhanced RFP (eRFP)-tagged wild-type (WT) PLK1 and S137D (SD), T210D (TD), S137D/T210D (SDTD), K82M (KM), and W414F/V415A (FA) mutants were expressed in NSCLC A549 and NCI-H460 cells. **a** A549 cells were selected with puromycin for 2 days and treated with doxycycline for 48 h. qRT-PCR was performed for *PLK1, CDH1, CDH2*, *VIM*, and *SNAI1* in A549 cells expressing various versions of PLK1. *p < 0.05; **p < 0.01; ***p < 0.001. (*n* = 3). Data presented as mean ± SD. **b** Immunoblot analyses were performed using anti-RFP, anti-PLK1, anti-E-cadherin, anti-N-cadherin, anti-snail, and anti-β-actin (upper panel). The band intensity values were quantified using LI-COR Odyssey software (Li-COR Biosciences), normalized, and plotted (lower panel). (*n* = 3). **c** Cell proliferation assay was performed. (*n* = 3). **d** A549 (upper panel) and NCI-H460 (lower panel) cells expressing various versions of PLK1 were subjected to a wound-healing assay, as shown in Supplementary Fig. S2. TGF-β was used as a positive control. The scratch recovery efficiency after 72 h was analyzed using NIS-Elements Imaging software (Nikon, Japan), and the relative migration distance compared with the control was plotted. *p < 0.05; **p < 0.01; ***p < 0.001. (*n* = 3). Data presented as mean ± SD. **e** A549 (upper panel) and NCI-H460 (lower panel) cells expressing various versions of PLK1 were subjected to a transwell migration assay. Three days after seeding, the cells on the bottom layer surface were stained with 0.05% crystal violet dye, and the intensity values were measured using an Odyssey infrared imaging system (LI-COR Biosciences) and plotted. *p < 0.05; **p < 0.01; ***p < 0.001. (*n* = 3). Data presented as mean ± SD. **f** A549 cells expressing various versions of PLK1 were subjected to an invasion assay. Seven days after seeding, the cells that had been invaded on the bottom layer surface were stained with 0.05% crystal violet dye, and the relative absorbance was plotted. (*n* = 3) Data presented as mean ± SD. **g** A colony formation assay was performed with A549 cells expressing various versions of PLK1, as described in the Materials and Methods. After 4 weeks, the colonies formed in agar were counted after 0.05% crystal violet staining. *p < 0.05; **p < 0.01; ***p < 0.001. (*n* = 3). Data presented as mean ± SD

The levels of PLK1 and p-PLK1 were high during TGF-β-induced EMT (Fig. 1f, g), so we next sought to determine whether PLK1 expression itself or its activity is more critical to inducing the EMT. In addition, we examined which functional domain, the catalytic domain or protein-interacting domain of PLK1, is involved in inducing the EMT (Supplementary Fig. S2A). For this investigation, a wild type of PLK1 (WT), a constitutively active phosphomimetic form at T210 (T210D; TD), a phosphomimetic form at S137 (S137D; SD), a double-phosphomimetic form at S137 and T210 (S137D/T210D; SDTD), a kinase-defective version (K82M; KM), and a variant with a mutation in the PBD (W414F/V415A; FA) were stably established in A549 cells using a doxycycline-inducible lentiviral system (Supplementary Fig. S2). The mRNA and protein levels of PLK1 in the cells expressing different versions of PLK1 were similar, as determined by qRT-PCR (Fig. 2a) and immunoblotting (Fig. 2b), respectively. In our experiments, the cells expressing WT had mRNA levels of the mesenchymal markers *CDH2* and *SNAI1* that were ~2-fold higher than the control. However, the mRNA levels of *CDH1*, an epithelial marker, were not much altered in WT–PLK1-expressing cells compared with the control (Fig. 2a). These patterns were dramatically visible in cells expressing TD, a constitutively active form PLK1 (Supplementary Fig. S2B). In cells expressing TD, the levels of *CDH2, VIM*, and *SNAI1* increased 3.2-, 2.1-, and 4.5-fold, respectively, compared with the control. On the other hand, *CDH1* levels decreased to around 0.7-fold in TD-expressing cells, compared with the control (Fig. 2a). Unlike TD, the expression of SD did not much affect the levels of *VIM* or *SNAI1*. However, expressing the KM or FA mutant reduced the expression of *CDH2* and *SNAI1* compared with the control (Fig. 2a). Consistent with the qRT-PCR data, the immunoblot analysis showed that the levels of N-cadherin and snail were higher in cells expressing TD, by ~3.5- and 3.2-fold, respectively, compared with the control (Fig. 2b). In cells expressing WT, the N-cadherin protein levels increased up to twofold compared with the control. The E-cadherin proteins in cells expressing TD or SDTD were lower by ~0.4- or 0.6-fold, respectively, compared with the control (Fig. 2b). The expression of SD, FA, or KM did not increase the levels of the mesenchymal markers, such as snail and N-cadherin, by very much. These data indicate that the catalytic activity of PLK1 is more critical to amplifying the mesenchymal transition than its expression (Fig. 2b). In other words, the expression of PLK1 increased the mesenchymal markers for the EMT, but they were more markedly enhanced by the expression of an active form of PLK1 phosphorylated at T210 than by the expression of PLK1 phosphorylated at S137, although their effects on cell proliferation were not much different (Fig. 2c). Instead, the cells expressing PLK1 phosphorylated at S137 showed epithelial traits, with high levels of E-cadherin and low levels of N-cadherin and snail. The ATP-binding domain for kinase activity is thus needed to accelerate the EMT. The PBD, a domain that interacts with phosphopeptides, had minimal effects on the EMT. Although the expression of WT-PLK1 induced the EMT, its kinase catalytic activity promoted the EMT more effectively than the expression of PLK1 itself. These data suggest that differential phosphorylation of PLK1 could function differentially in cells with mesenchymal and epithelial status.

### Active form of PLK1 accelerates cancer cell motility, invasiveness, and tumorigenicity

To functionally examine the effects of the PLK1 domains on cell motility, we performed a wound-healing assay and a transwell cell-migration assay. The wound-healing assay was performed in NSCLC A549 and NCI-H460 cells 24, 48, and 72 h after expressing the WT, SD, TD, SDTD, KM, and FA mutants of PLK1. Images of the wounded monolayers were collected and analyzed with a microscope (Fig. 2d and Supplementary Fig. S3). The motility of the A549 cells increased in cells expressing WT, SD, TD, and SDTD, compared with the control. In particular, the motility of cells expressing TD increased dramatically, by 91% compared with the control when the control motility 72 h after wounding was defined as 0% (Fig. 2d upper panel; Supplementary Fig. S3). The cell motility of SD was similar to that of WT at 72 h, suggesting that phosphorylation at S137 does not much affect the cell migration driven by PLK1 expression. In cells expressing KM or FA, cell motility was lower by ~24.8% and 12.7%, respectively, compared with the control, indicating that the distance between the cells expressing KM or FA was farther than that of the control (Fig. 2d, upper panel and Supplementary Fig. S3A). Consistently, NCI-H460 cell motility was increased by expressing TD and reduced by expressing KM or FA (Fig. 2d, lower panel and Supplementary Fig. S3B). The motility of cells expressing active PLK1 was the highest, with 31% of the relative migration distance when the motility of the control 72 h after wounding was defined as 0%. On the other hand, the motility of cells expressing the inactive forms, KM and FA, was lower by ~31.8% and 14.7%, respectively, compared with the control (Fig. 2d). Consistent with the changes in EMT marker expression, the catalytic activity of PLK1 is important to PLK1-induced cell motility.

A transwell cell-migration assay was performed in primary A549 cell and metastatic NCI-H460 cells to confirm that their motility was altered by expressing the different PLK1 variants (Fig. 2e). Consistent with the wound-healing analysis, the motility of A549 and NCI-H460 cells was the greatest when they were expressing active PLK1. The motility of cells expressing SD did not differ much from that of WT, indicating that phosphorylation of PLK1 at S137 does not influence cell motility (Fig. 2e). Furthermore, the relative motilities of A549 cells expressing the inactive KM and FA forms were lower by ~0.69 and 0.83, respectively, compared with the control, when the relative motility of the control was defined as 1. (Fig. 2e). Consistent with the A549 results, the relative motility of the NCI-H460 cells expressing the inactive KM and FA forms was lower than that of the control by ~0.76 and 0.88, respectively (Fig. 2e). Thus, active PLK1 promoted cell motility, whereas the kinase-defective mutant and PBD mutant did not, indicating that the catalytic activity of PLK1 and its interaction with phosphopeptides are important to the cell motility driven by PLK1.

Invasiveness is the main characteristic of cancer and the first step of metastasis. To investigate whether the active form of PLK1 triggers invasiveness in cancer, we performed an invasion assay using Matrigel (Fig. 2f). A549 cells expressing WT or mutants of PLK1 were layered in a Matrigel insert and treated with TGF-β, and then crystal violet was used for staining (Fig. 2f). The highest absorbance was shown in cells expressing the catalytically active TD, and the lowest was in cells expressing the inactive KM or FA (Fig. 2f). Thus, the active version of PLK1 promotes the invasiveness of cancer cells as well as cell motility.

We also evaluated whether the active form of PLK1 triggers the tumorigenicity of cancer. For this investigation, we performed a colony formation assay (Fig. 2g). The colonies formed by cells expressing the WT or mutant types of PLK1 were observed for 4 weeks, using 0.6 and 0.4% agarose for a soft agar colony formation assay (Fig. 2g). The greatest number of colonies was found in A549 cells expressing TD, and the lowest number was in cells expressing KM or FA. Taken together, our results show that the expression of active PLK1 increased the cell motility, invasiveness, and tumorigenicity of cancer cells, whereas the expression of a catalytically inactive version or a version with a non-functional PBD blocked those abilities, indicating that PLK1 must have catalytic activity or protein-binding ability to amplify tumorigenic and metastatic progression.

### Loss of PLK1 activity blocks pro-tumorigenic and pro-metastatic activity induced by TGF-β or PLK1

**Fig. 3:**
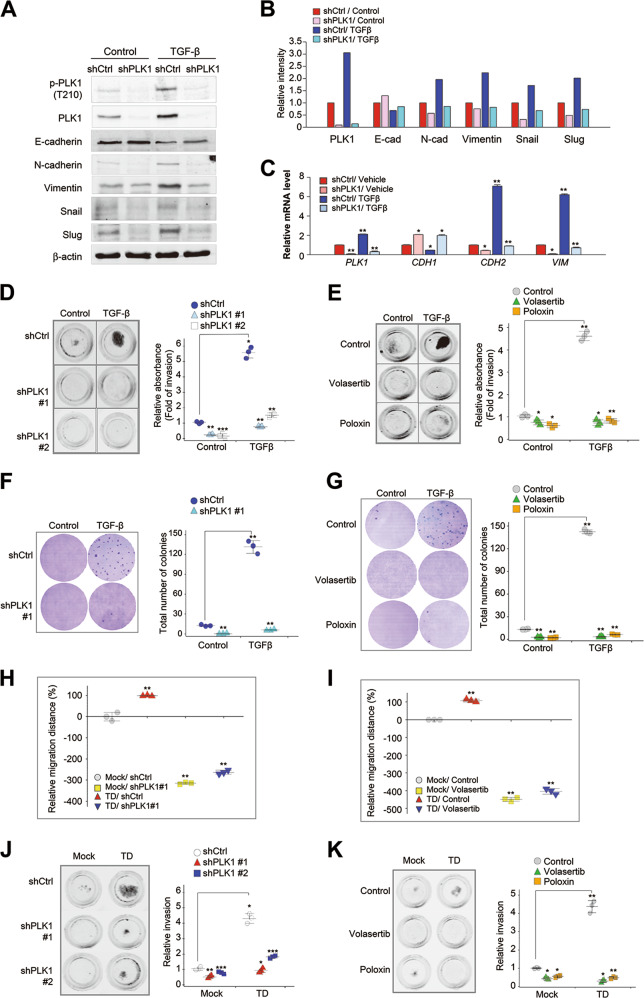
Loss of PLK1 activity blocks pro-tumorigenic and pro-metastatic activity induced by active PLK1 or TGF-β. **a**–**c** A549 cells were infected by lentiviral PLK1 shRNA #1 and then treated with TGF-β for 48 h. **a** Immunoblot analyses were performed using anti-PLK1, anti-pT210-PLK1, anti-N-cadherin, anti-E-cadherin, anti-vimentin, anti-snail, anti-slug, and anti-β−actin. **b** The band intensity values were quantified using LI-COR Odyssey software (Li-COR Biosciences), normalized, and plotted. **c** qRT-PCR was performed for *PLK1, CDH1, CDH2*, and *VIM* in A549 cells with depleted PLK1. *p < 0.05; **p < 0.01; ***p < 0.001 (*n* = 3). Data presented as mean ± SD. **d** A549 cells were infected by lentiviral PLK1 shRNA #1 (shPLK1#1) and #2 (shPLK1#2) and then treated with TGF-β. The cells that had been invaded on the bottom layer surface were stained with 0.05% crystal violet dye, and the relative absorbance was plotted. (*n* = 3). **e** An invasion assay was performed in TGF-β-treated A549 cells after treatment with the PLK1 inhibitors volasertib and poloxin for 5 days. The absorbance was measured at a wavelength of 590 nm, and the relative absorbance was plotted. (*n* = 3). Data presented as mean ± SD. **f** A colony formation assay was performed with A549 cells depleted of PLK1, as described in the Materials and Methods. After 4 weeks, the colonies formed in agar were counted after 0.05% crystal violet staining. *p < 0.05; **p < 0.01; ***p < 0.001. (*n* = 3). Data presented as mean ± SD. **g** A colony formation assay was performed in TGF-β-treated A549 cells after treatment with the PLK1 inhibitors volasertib and poloxin. After 4 weeks, the colonies formed in agar were counted after 0.05% crystal violet staining. *p < 0.05; **p < 0.01; ***p < 0.001. (*n* = 3). Data presented as mean ± SD. **h**–**i** A549 cells expressing eRFP-tagged TD were treated with **h** shRNA targeting human PLK1 or **i** volasertib, and an in vitro wound-healing assay was performed 72 h after treatment. The relative migration distance was measured and plotted. *p < 0.05; **p < 0.01; ***p < 0.001. (*n* = 3). **j**–**k** An invasion assay was performed using A549 cells expressing eRFP-tagged TD after treatment with **j** shRNA for PLK1 #1 or #2 **k** volasertib and poloxin. The relative absorbance was measured and plotted. *p < 0.05; **p < 0.01; ***p < 0.001. (*n* = 3)

As we found that functionally intact PLK1 was required to accelerate tumorigenesis and metastasis (Fig. 2), we performed loss of function experiments to clarify whether PLK1 is required for TGF-β-induced EMT in NSCLC. We infected A549 cells using lentiviral short-hairpin RNA (shRNA) targeting of human PLK1 and treated with TGF-β for proliferation and migration assay (Supplementary Fig. S4, A, B). Cell migration was markedly reduced by treatment of shPLK1#1 (targeting 1424–1624) or #2 (targeting 245–265) (Supplementary Fig. S4, A, B). Immunoblot and qRT-PCR analyses showed that the expression and phosphorylation of PLK1 were blocked by the depletion of PLK1 using shPLK1#1, regardless of the presence of TGF-β–induced EMT signaling (Fig. 3a–c). The increased levels of N-cadherin and vimentin caused by the treatment with TGF-β were reduced by PLK1 depletion (Fig. 3a–c). In addition, the reduced levels of E-cadherin caused by treatment with TGF-β were weakly rescued by PLK1 depletion (Fig. 3a–c). Thus, PLK1 knockdown markedly suppressed TGF-β-induced EMT.

Further studies were performed to determine whether the depletion of PLK1 or the inhibition of its activity could block the cell invasiveness induced by treatment with TGF-β. We performed a transwell-based invasion assay with shRNA targeting PLK1 (#1 and #2) or PLK1-specific inhibitors (volasertib and poloxin) (Fig. 3d, e). In the transwell-based invasion assay, treatment with 2.5 ng/ml TGF-β increased the number of invasive cells, which was markedly reduced by depleting PLK1 using shRNA or the PLK1 inhibitors volasertib or poloxin, although cells were still proliferated under the conditions (Fig. 3d, e and Supplementary Fig. S4, A–C). In addition, a colony formation assay was performed to determine whether the absence of PLK1 or the inhibition of its activity could block the tumorigenicity induced by TGF-β (Fig. 3f, g). When A549 cells were cultured with TGF-β in agar, more than 130 cell colonies were formed vs. ~10 colonies in the control shRNA condition. Knockdown of PLK1 with shRNA markedly suppressed colony formation, with fewer than five colonies in the TGF-β-treated condition (Fig. 3f). In addition, PLK1 activity was inhibited using volasertib or poloxin, which also suppressed the colony formation triggered by TGF-β (Fig. 3g). Thus, the expression of PLK1 and its activity are both critical to inducing cell invasiveness and tumorigenicity in NSCLC cells.

Next to investigate whether metastatic activity driven by active PLK1, can be blocked by the depletion or inhibition of PLK1. Using PLK1 shRNA or volasertib, we performed a wound-healing assay and a transwell-based invasion assay (Fig. 3h–k). The depletion of PLK1 using lentiviral shRNA #1 reduced the cell motility induced by the expression of TD 72 h after depletion (Fig. 3h and Supplementary Fig. S4D). The motility of cells expressing TD was ~99% that of the control 72 h after scratching, but the motility of cells treated with PLK1 shRNA was markedly reduced (Fig. 3h). In addition, blocking the activity of PLK1 with volasertib reduced cell motility by ~400% compared with the control (Fig. 3i and Supplementary Fig. S4E). These data show that the increased motility induced by active PLK1 was reduced by blocking PLK1 expression or inhibiting its activity. To determine whether the inhibition of PLK1 activity could block cell invasiveness, we performed a transwell-based invasion assay with shRNA (#1 and #2) and the PLK1-specific inhibitors volasertib and poloxin in cells expressing TD-PLK1 (Fig. 3j, k). We found that the expression of TD increased the number of invading cells, which was markedly reduced by depleting PLK1 using shRNA (Fig. 3j) or treatment with volasertib or poloxin (Fig. 3k), as determined by the absorbance at 590 nm. These data indicate that PLK1 activity is essential to inducing metastasis and that it can effectively be blocked by the specific inhibitor volasertib.

### Active PLK1 promotes metastasis in tail-vein injection mouse model

**Fig. 4:**
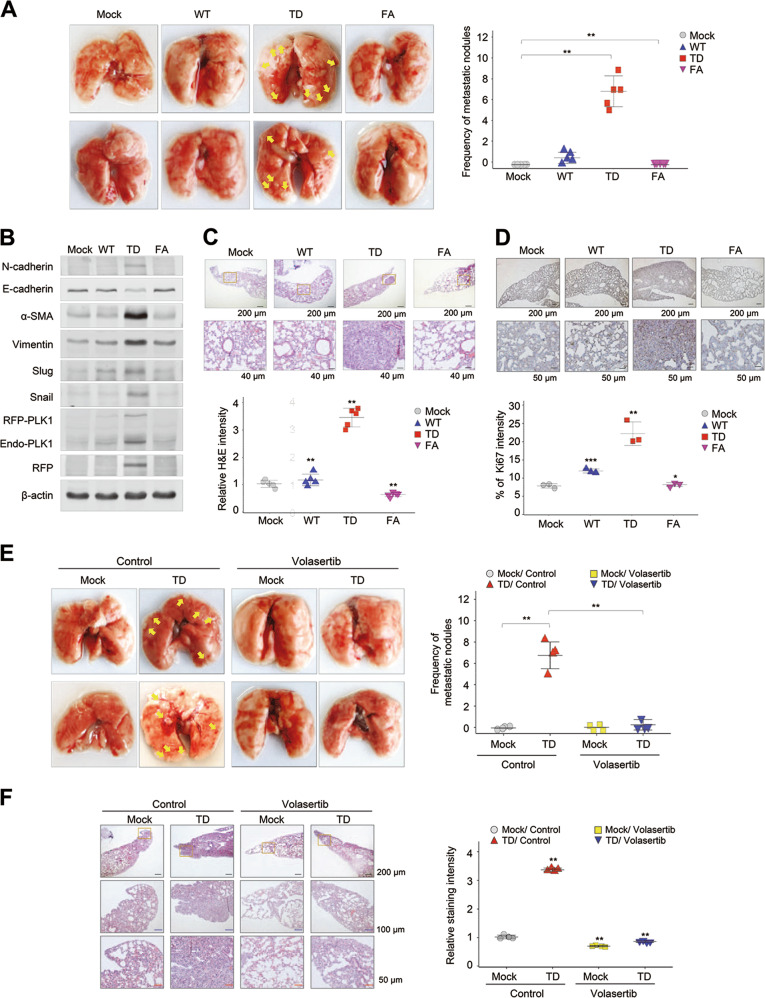
Expression of active PLK1 promotes tumor growth and metastasis in mouse model, which is blocked by the treatment of volasertib. **a**–**d** A549 cells expressing phosphomimetic active T210D (TD) or inactive PBD mutant W414F/V415A (FA) PLK1 were injected intravenously into the tail-veins of 4-week-old BALB/c nude mice, and the tumorigenic and metastatic properties were evaluated after 10 weeks. **a** Representative lung tumors from the mouse model (left panel). The number of metastatic lung tumors was counted and plotted (right panel) (*n* *=* 5). Data presented as mean ± SD. **b** Immunoblotting was performed using lung tissue lysates from each mouse model. N-cadherin, E-cadherin, α-SMA, vimentin, slug, snail, PLK1, RFP, and β-actin were detected using specific antibodies. **c** H&E staining was performed using lung tissue from the mice. *p < 0.05; **p < 0.01; ***p < 0.001. (*n* = 3). Data presented as mean ± SD. **d** Ki-67 staining was performed using lung tissue from the mice. *p < 0.05; **p < 0.01; ***p < 0.001. (*n* = 3). Data presented as mean ± SD. **e**–**f** Primary A549 cells expressing phosphomimetic active TD-PLK1 were injected intravenously into the tail veins of 4-week-old BALB/c nude mice. Two weeks later, the mice received 20 mg/kg of volasertib by injection every week for 3 weeks. After 5 weeks, the anti-tumorigenic and anti-metastatic properties of volasertib were evaluated. **e** Representative lung tumors from the mouse model (left panel). The number of metastatic lung tumors was counted and plotted (right panel) (*n* ≥ 4). **f** H&E staining was performed using lung tissue from the mice. The relative intensity of the H&E staining was plotted. *p < 0.05; **p < 0.01; ***p < 0.001. (*n* = 4). Data presented as mean ± SD

To explore the effects of active PLK1 on tumorigenesis and metastasis in an in vivo model, primary A549 cells expressing phosphomimetic active TD-PLK1 or the inactive PBD mutant FA-PLK1 were injected intravenously into the tail-veins of BALB/c nude mice. We observed whether the expression of active PLK1 enhanced metastasis and tumor formation (Fig. 4a and Supplementary Fig. S5). Ten weeks after the injection, tumors were found in the lungs through blood-circulation, with more than five nodules in each mouse (*n* = 5) whose cells expressed active PLK1 (TD). In the mice with cells expressing WT-PLK1, tumors formed in 40%, with one nodule in each mouse. On the other hand, no metastatic nodules were observed in any of the mice injected with cells expressing FA-PLK1 (Fig. 4a). We performed immunoblot analyses of lung tissue or metastatic lung cancer tissue from each mouse to evaluate whether cells expressing PLK1 had metastasized to the lung and if so, which factors were expressed in the metastatic cancer. We found that exogenous RFP-tagged PLK1 proteins were dominantly higher in metastatic lung cancer expressing TD than in lung tissue expressing WT or FA (Fig. 4b). In our experiments, the protein levels of the mesenchymal markers (N-cadherin, vimentin, α-SMA, snail, and slug) were higher in the lung tissue expressing TD than in that expressing mock, WT, or FA. In addition, the levels of E-cadherin were reduced in metastatic lung cancer expressing TD (Fig. 4b). In hematoxylin and eosin (H&E) and Ki-67 staining, the relative intensities of H&E and Ki-67-positive cells in lung tissue expressing TD were higher than those expressing mock, WT, or FA, evidence of TD’s tumorigenic properties in the in vivo metastatic mouse model (Fig. 4c, d), suggesting that catalytically active PLK1 promotes metastasis and tumorigenicity in vivo and that non-functional mutants of PLK1 lose those tumorigenic properties. Thus, the presence of the functional domain of PLK1 is important in inducing cancer metastasis and tumorigenicity, which are accelerated by active PLK1 in NSCLC.

Next, the blocking effects of the PLK1 inhibitor volasertib on metastasis and tumorigenicity were observed in vivo. Mice received intravenous injections of A549 cells expressing TD into their tail veins, and after 2 weeks, 20 mg/kg of volasertib was intravenously injected every week for 3 weeks (Fig. 4e). After a total of 10 weeks, the frequency of metastatic lung nodules induced by the expression of active PLK1 was suppressed by the volasertib injections (Fig. 4e). In addition, the relative intensity of H&E staining of cells expressing TD was suppressed by the volasertib treatment (Fig. 4f). Taken together, our results show that PLK1 activity is essential to inducing tumor formation and metastasis and that it can effectively be blocked by the specific inhibitor volasertib in an animal model, as well as in cell-based experiments, suggesting that PLK1 is an effective target for treating cancer metastasis.

### TGF-β signaling is upregulated in PLK1-driven invasive cells

**Fig. 5:**
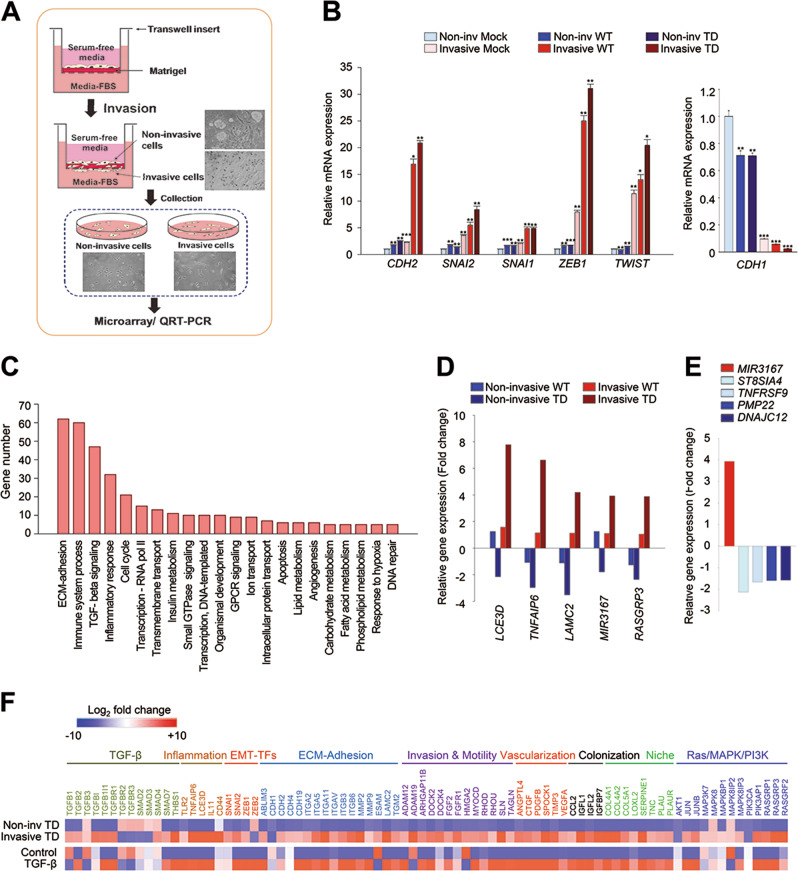
TGF-β signaling is upregulated in PLK1-driven invasive cells. **a** Three-dimensional culture scheme for invasive cells. **b** qRT-PCR was performed for *CDH1, CDH2*, *SNAI1, SNAI2, ZEB1*, and *TWIST* in invasive and non-invasive A549 cells expressing WT or TD-PLK1. *p < 0.05; **p < 0.01; ***p < 0.001. **c** Analysis of transcriptome data for gene probes with significant fold changes (>1.5) in the invasive cells expressing TD. The significant genes were categorized using the KEGG pathway. The top 20% of the 117 pathways are displayed. **d** Relative gene expression profile of the top five genes in invasive and non-invasive cells expressing WT and TD, respectively. **e** Relative gene expression profile of the targets of miRNA 3167 (*ST8SIA4*, *TNFRSF9*, *PMP22*, and *DNAJC12*) in invasive cells expressing TD. **f** Transcriptome comparison between the gene profiles of invasive and non-invasive A549 cells expressing TD and gene profiles of TGF-β-induced mesenchymal A549 cells (GSE 46024). The MORPHEUS program was used to visualize the expression levels of genes related to TGF-β signaling, Ras/MAPK/PI3K signaling, transcriptional factors of the EMT, ECM-adhesion, invasion, motility, vascularization, colonization, and niche in non-invasive and invasive A549 cells expressing active PLK1 (TD) and in the published transcriptome of TGF-β-treated A549 cells (GSE 46024)

We next investigated which factors are triggered and involved in PLK1-driven metastasis by collecting cells expressing TD in a transwell-based invasion assay 10 days after seeding (Fig. 5a). The cells that had been invaded attached to the outer surface, and the cells that had not been invaded attached to the inner well; they were all treated with trypsin and then prepared for qRT-PCR and transcriptome profiling. The qRT-PCR analysis revealed that the levels of *CDH1* were higher in the cells that had not been invaded than in those that had (Fig. 5b, left panel). The levels of the mesenchymal markers (*CDH2, SNAI1, SNAI2, ZEB1*, and *TWIST*) were highly upregulated in the cells that had been invaded compared with those that had not (Fig. 5b, right panel). Again, we observed that the expression of active PLK1 enhanced lung cell invasiveness, producing PLK1-driven metastasis. We performed a microarray analysis for transcriptome profiling to define which factors can be affected and expressed by PLK1-driven invasiveness and metastasis. As PLK1-driven metastasis in cells expressing TD was much greater than in those expressing WT, the genes of invasive cells expressing TD were analyzed (Fig. 5c). In the transcriptome data, we clustered gene probes with fold changes >1.5 (Fig. 5c and Supplementary Table S1), which revealed that the levels of 663 genes were changed significantly in the invasive cells expressing TD (Fig. 5c). After excluding unknown factors and RNA, we categorized the remaining 472 genes using the KEGG pathway. The top 20% of the 117 pathways (472 genes) are displayed in Fig. 5c. Our analysis revealed that genes related to ECM-adhesion, immune response, TGF-β-signaling, inflammation, and the cell cycle were the main ones altered in the TD-induced invasive cells (Fig. 5c). Of the top five genes highly expressed in the active PLK1-driven invasive cells, *LCE3D, TNFAIP6, LAMC2*, and *RASGRP3* are related to TGF-β-signaling or an inflammatory response pathway (Fig. 5d). MiRNA 3167 was highly expressed in TD-driven invasive cells. Since miRNA 3167 downregulates its targets such as *ST8SIA4*, *TNFRSF9*, *PMP22*, and *DNAJC12* in the previous report [28], their levels were analyzed using our microarray data. As a response to miRNA 3167 upregulation, *ST8SIA4*, *TNFRSF9*, *PMP22*, and *DNAJC12*, were downregulated >1.5-fold in the active PLK1-driven invasive cells (Fig. 5e and Supplementary Table S2). In addition, the gene changes were analyzed in both invasive and non-invasive TD-expressing cells (Fig. 5f), which revealed that TD-driven invasion triggers the expression of genes related to EMT, including TGF-β signaling, PI3K-Akt, ECM-receptor interaction, invasion, and motility. We next compared those data with the transcriptome of TGF-β-induced mesenchymal cells and found that genes related to TGF-β signaling were expressed more highly in TGF-β-treated A549 cells than in control cells (GSE 46024) [29]. The expression profiles between TGF-β-treated A549 cells and the invasive cells induced by expressing TD-PLK1 shared similar patterns (Fig. 5f), suggesting that active PLK1-driven metastasis could be amplified by genes related to TGF-β-signaling.

### Depletion of *TNFAIP6* suppresses the cell migration and invasion induced by active PLK1 expression or TGF-β treatment

**Fig. 6:**
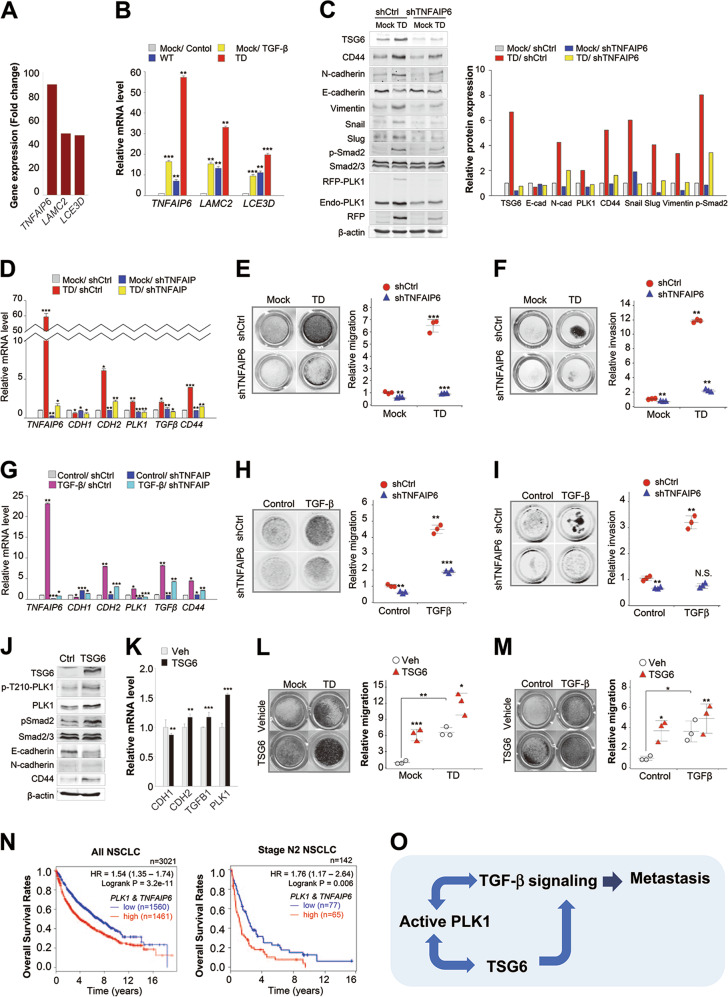
Depletion of *TNFAIP6* reduces the metastatic activity induced by active PLK1 or TGF-β in NSCLC. **a** Relative gene expression profile of top three genes in invasive cells expressing TD, compared with non-invasive cells expressing TD. **b** qRT-PCR was performed for *TNFAIP6, LAMC2*, or *LCE3D* of invasive A549 cells expressing mock, WT, or TD-PLK1. TGF-β was used as a positive control for the EMT. **p < 0.01; ***p < 0.001. (*n* = 3). Data presented as mean ± SD. **c–f** A549 cells expressing mock or TD-PLK1 (TD) were depleted with shRNA targeting *TNFAIP6*. **c** Immunoblotting was performed using specific antibodies for TSG6, CD44, N-cadherin, E-cadherin, vimentin, snail, slug, smad2/3, p-smad2, PLK1, RFP, and β-actin (left panel). The band intensity values were quantified with LI-COR Odyssey software, normalized, and plotted (right panel). **d** qRT-PCR was performed for *TNFAIP6, CDH1, CDH2, TGFB1*, or *CD44* in A549 cells expressing mock or TD-PLK1 depleting with shRNA targeting for *TNFAIP6*. *p < 0.05; **p < 0.01; ***p < 0.001. (*n* = 3). Data presented as mean ± SD. **e** A549 cells were subjected to a transwell migration assay. Three days after seeding, the cells on the bottom layer surface were stained with 0.05% crystal violet dye, and the intensity values were measured using an Odyssey infrared imaging system (LI-COR Biosciences) and plotted. **p < 0.01; ***p < 0.001. (*n* = 3). **f** A549 cells expressing mock or TD-PLK1 were de*p*leted with shRNA targeting *TNFAIP6* and subjected to an invasion assay. Seven days after seeding, the cells that had been invaded on the bottom layer surface were stained with 0.05% crystal violet dye, and the relative invasion was plotted. *p < 0.05; **p < 0.01; ***p < 0.001. (*n* = 3). **g–i** A549 cells depleted of *TNFAIP6* were treated with TGF-β for 48 h. **g** qRT-PCR was performed for *TNFAIP6, CDH1, CDH2, TGFB1*, or *CD44*. *p < 0.05; **p < 0.01; ***p < 0.001. (*n* = 3). **h** A549 cells depleting *TNFAIP6* were treated with TGF-β and subjected to a transwell migration assay. Three days after seeding, the cells on the bottom layer surface were stained with 0.05% crystal violet dye, and the intensity values were measured using an Odyssey infrared imaging system (LI-COR Biosciences) and plotted. **p < 0.01; ***p < 0.001. (*n* = 3). **i** A549 cells depleted of *TNFAIP6* were treated with TGF-β and subjected to an invasion assay. Five days after seeding, the cells that had been invaded on the bottom layer surface were stained with 0.05% crystal violet dye, and the relative invasion was plotted. *p < 0.05; **p < 0.01; ***p < 0.001. (*n* = 3) NS, not significant. **j** TSG6-treated A549 cells were subjected to immunoblot analysis using specific antibodies for TSG6, PLK1, p-T210-PLK1, N-cadherin, E-cadherin, smad2/3, p-smad2, and β-acti*n*. (*n* = 3). **k** qRT-PCR was performed for *PLK1, CDH1, CDH2*, or *TGFB1* in TSG6-treated A549 cells. **p < 0.01; ***p < 0.001. (*n* = 3). Data presented as mean ± SD. **l** A549 cells expressing TD-PLK1 were treated with TSG6 and subjected to a transwell migration assay. The cells were stained with 0.05% crystal violet dye, and the intensity values were measured and plotted. **p < 0.01; ***p < 0.001. (*n* = 3). **m** A549 cells treating TGF-β were treated with TSG6 and subjected to a transwell migration assay. *p < 0.05; **p < 0.01. (*n* = 3). **n** Clinical association between *PLK1* and *TNFAIP6* in patients with lung cancer. The survival rate of NSCLC patients was analyzed according to the *PLK1* and *TNFAIP6* expression levels using KM PLOTTER in all NSCLC patients (*n* = 3021) or stage N2 NSCLC patients (*n* = 142). **o** Plausible model of PLK1-driven metastasis

The relative gene changes in invasive TD-expressing cells compared with non-invasive TD, revealed that *TNFAIP6, LAMC2*, and *LCE3D* ranked within top three genes highly expressed in the invasive TD-expressed cells (Fig. 5f and Supplementary Fig. S6). To confirm the correlation between the presence of active PLK1 and the gene expression of *TNFAIP6*, *LCE3D*, and *LAMC2*, the top three genes highly expressed in active PLK1-driven invasive cells (Fig. 6a), we performed qRT-PCR of A549 cells expressing active PLK1 or treated with TGF-β (Fig. 6b). The level of *TNFAIP6* mRNA was markedly upregulated, up to ~60-fold, in active PLK1-expressing cells compared with the control cells. *LCE3D* and *LAMC2* mRNA were also highly expressed in PLK1-expressing cells, as expected (Fig. 6b). In TGF-β-treated A549 cells, the mRNA levels of *TNFAIP6* increased ~15-fold compared with the control. Compared with TGF-β-induced EMT cells, the expression of active PLK1 upregulated the expression of *TNFAIP6* by ~3-fold (Fig. 6b). Thus, the presence of active PLK1 is associated with the upregulation of *TNFAIP6*.

As *TNFAIP6* showed the highest genetic alterations among the top three genes in TD-expressing cells, we focused on the function of *TNFAIP6*. To investigate whether *TNFAIP6* could trigger the metastatic properties of NSCLC, active PLK1-expressing cells were treated with shRNA for *TNFAIP6* (Fig. 6c, d). Immunoblot analysis (Fig. 6c) and qRT-PCR (Fig. 6d) revealed that the depletion for *TNFAIP6* suppressed the EMT induced by the expression of active PLK1, as determined by *CDH1* (E-cadherin) and *CDH2* (N-cadherin) levels (Fig. 6c, d). The levels of additional mesenchymal markers (vimentin, snail, and slug) and the phosphorylation of Smad2, the TGF-β effector, were elevated in cells expressing active PLK1, whereas depletion of *TNFAIP6* reduced their expressions (Fig. 6c). The expression of the lung cancer stem cell marker *CD44* and TGF-β ligand *TGFB1* was also reduced by the depletion of *TNFAIP6* in active PLK1-expressing cells. Next, we wanted to determine whether the suppression of *TNFAIP6* could block cell migration and invasiveness in active PLK1-driven metastatic cancer. Therefore, we performed a transwell-based migration assay and an invasion assay in cells depleted of *TNFAIP6* (Fig. 6e, f). We found that depleting *TNFAIP6* using shRNA markedly reduced the increased number of migrating and invasive cells in PLK1-TD-expressing A549 cells (Fig. 6e, f). Thus, depletion of *TNFAIP6* suppressed the active PLK1-driven migration and invasiveness.

Next, to evaluate whether the effects of *TNFAIP6* depletion on the EMT are general phenomenon, an EMT inducer TGF-β was used (Fig. 6g–i). Treatment of TGF-β upregulated mRNA levels of *PLK1, CD44*, and *TNFAIP6* (Fig. 6g). qRT-PCR revealed that depletion for *TNFAIP6* suppressed the mRNA levels of *CDH2*, *TGB1*, and *CD44*, induced by TGF-β treatment (Fig. 6g). In addition, the suppression of *TNFAIP6* blocked cell migration and invasiveness in TGF-β-induced metastatic A549 cells, as determined by a transwell-based migration assay (Fig. 6h) and an invasion assay (Fig. 6i). These data indicate that depletion of *TNFAIP6* suppressed TGF-β-induced EMT reprogramming, cell migration, and invasiveness. To gain a complete understanding whether TSG6 can trigger EMT mediated by PLK1 activation and TGF-β signaling, immunoblotting and qRT-PCR were performed in TSG6-treated A549 cells (Fig. 6j, k). TSG6 treatment upregulated the levels of p-T210-PLK1 and p-Smad2, and the mRNA levels of PLK1 (Fig. 6j, k). Moreover, TSG6 treatment accelerated cell migration induced by expressing active TD or treatment with TGF-β (Fig. 6l, m). These data indicate that TSG6 triggers EMT through the activation of PLK1 and TGF-β signaling.

To explore further the clinical relevance of the expressions of *PLK1* with *TNFAIP6* to survival rates in metastatic NSCLC, we analyzed OS (Fig. 6n). The OS rate of NSCLC patients with high expression of *PLK1* with *TNFAIP6* was lower than that of NSCLC patients with low expression of *PLK1* with *TNFAIP6* (*n* = 3021, HR = 1.54, log rank *p* = 3.2e-11) with high significance (Fig. 6n, left panel). In a clinical analysis of 142 metastatic NSCLC patients with AJCC stage N2, the significance remained in the analysis of *PLK1* with *TNFAIP6* (Fig. 6n, right panel). Metastatic AJCC stage N2 patients with high levels of *PLK1* and *TNFAIP6* had lower OS rates than patients with low *PLK1* and *TNFAIP6* expression (*n* = 142, HR = 1.76, log rank *p* = 0.006) (Fig. 6n, right panel). Combining *PLK1* and *TNFAIP6* has a more significant prognostic values than either gene alone, especially in stage N2 NSCLC (Figs. 6n and 1b; and Supplementary Fig. S7). Thus, high expression of both *TNFAIP6* and *PLK1* would be a negative marker for survival in primary or metastatic NSCLC patients.

## Discussion

In this study, we have demonstrated five main findings about PLK1-driven EMT and metastasis. First, the active form of PLK1 phosphorylated at T210 is abundant in TGF-β-induced metastatic NSCLC, and the presence of active PLK1 promotes metastasis of NSCLC in vivo. The expression of phosphomimetic PLK1 at T210 accelerated invasiveness and metastasis, but that at S137 did not. The expression of different phosphomimetic mutants of PLK1 produced different phenotypes in epithelial and mesenchymal characters, suggesting that the differential phosphorylation of PLK1 could function differentially in mesenchymal and epithelial status. Second, PLK1 activity is essential for pro-tumorigenic and pro-metastatic activity in three-dimensional (3D) culture and an in vivo metastatic mouse model. Loss of PLK1 activity through the use of specific inhibitors or shRNA blocked pro-tumorigenic and pro-metastatic activity in 3D culture and the in vivo model, indicating that blocking PLK1 activity suppressed PLK1-driven metastasis and tumorigenicity. Third, the invasiveness and metastasis driven by active PLK1 were amplified by TGF-β signaling. The invasiveness induced by expressing active PLK1 upregulated the levels of p-Smad2, the TGF-β effector, and the expression of several genes related to TGF-β signaling, including *SNAI1, SNAI2, ZEB1, CDH2, IL11*, and *TNFAIP6*. Fourth, the activation of TGF-β signaling through the expression of active PLK1 upregulates *TNFAIP6*, which promotes metastasis and invasiveness in NSCLC because *TNFAIP6* is induced by several growth factors, including TGF-β [30, 31]. The depletion of *TNFAIP6*, top-ranked gene expressed highly in invasive cells expressing active PLK1, disturbed cell migration, invasion, and the EMT induced by active PLK1 or TGF-β. Fifth, *TNFAIP6* and *PLK1* are strong predictors of poor survival rates in metastatic NSCLC patients. Clinical data provide evidence for the connection between the expression of *PLK1* and *TNFAIP6* on the one hand and the OS rates of NSCLC metastatic patients on the other. Metastatic AJCC stage N2 patients with high levels of *PLK1* and *TNFAIP6* had lower survival rates than similar patients with low *PLK1* and *TNFAIP6*, suggesting that the high expression of both *TNFAIP6* and *PLK1* is a negative marker for survival among primary and metastatic NSCLC patients.

Clinically, PLK1 has been raised as a target for cancer treatment because of its connection with malignancy and advanced cancer development in NSCLC [14, 32, 33]. Our clinical relevance analysis revealed that the expression of PLK1 could be useful in diagnosing NSCLC patients with a high risk of metastasis because PLK1 levels were higher in patients with metastatic stage N2 NSCLC and low survival rates (Fig. 1b). For the clinical analysis of metastatic NSCLC patients, we used data from NSCLC patients with metastases to ipsilateral, mediastinal, or subcarinal lymph nodes at stage N2 of the TMN classification because the population of NSCLC patients with distant metastases (AJCC stage M) or AJCC stage N3 was too small to generate a KM plot. As shown by our analysis, PLK1 can be used clinically as an index of malignancy and advanced metastatic NSCLC.

Our in vivo metastatic animal model using tail-vein injection supported the evidence that active PLK1 induces the EMT and metastasis. Although previous studies of PLK1 function in EMT had been limited to cell culture systems [17–19], we here used an in vivo metastatic animal model in addition to a 3D-culture system. In mice with cells expressing the active form of PLK1, tumors were found in the lung through blood-circulation, with more than five nodules in each mouse. On the other hand, no metastatic nodules were observed in any of the mice injected with cells expressing mock or polo-box mutant PLK1. The frequency of metastatic lung nodules induced by the expression of active PLK1 was suppressed by injection with the PLK1 inhibitor volasertib. Immunoblot analyses of lung tissue or metastatic lung cancer tissue from each mouse revealed that mesenchymal markers, including N-cadherin, vimentin, α-SMA, snail, and slug, were increased in lung tissue expressing TD compared with those expressing mock, WT, or FA. These data suggest that catalytically active PLK1 promotes metastasis and tumorigenicity in vivo and that non-functional mutants of PLK1 lose their tumorigenic properties. Thus, PLK1 activity is needed to induce tumor formation and metastasis, which was effectively blocked by specific the inhibitor volasertib in both an animal model and our cell-based 3D-culture system.

The activation of the TGF-β signaling pathway induces the EMT and invasiveness by decreasing the levels of the epithelial index E-cadherin and increasing the expression of the mesenchymal proteins N-cadherin and vimentin [7, 34]. In the tumor microenvironment, TGF-β signaling has various effects for tumor progression and metastasis by phosphorylating or interacting with numerous factors, depending on the cell context [8–10]. Transcriptome profiling of invasive cells expressing active PLK1 revealed that the main alternations are in genes related to cell proliferation and the EMT process, including ECM-adhesion, TGF-β ligands/receptors, and Smad, PI3K/Akt, and Ras/MAPK signaling. Among the top five genes expressed highly in active PLK1-driven invasive cells, *TNFAIP6, LAMC2*, *LCE3D*, and *RASGRP3* are involved in TGF-β-related responses or the inflammatory response pathway (Fig. 5d). Active PLK1-driven invasiveness induces transcriptional reprogramming, which is amplified by TGF-β signaling and *TNFAIP6*.

*TNFAIP6* was detected as the most highly expressed gene in active PLK1-expressing invasive A549 cells. TNFα-stimulated gene 6 (TSG6) encoded by *TNFAIP6* is known as a hyaluronan (HA)-binding protein and involved in extracellular matrix stability, the EMT process, and cell migration [30, 35]. It also activates macrophage in response to the inflammatory injury [36]. It was reported that TSG6 functions in the EMT of proximal tubular epithelial cells through effects on HA structure and CD44-dependent triggering of cell responses [35]. CD44 is closely related to TSG6 as an HA receptor [37] and has been studied as a cancer stem cell marker in lung cancer [38]. It was reported that CD44 also contributes to fibroblast migration through the activation of TGF-β [39]. The evidence supports that TSG6 and CD44 contribute cell migration through their interactions with ECM-adhesion molecules in response to TGF-β signaling. In our results, changes to TSG6 and CD44 were directly affected by the EMT induced by active PLK1 expression or TGF-β treatment. The regulatory mechanisms of TSG6 and CD44 in cancer metastasis still have to be investigated. Loss of TSG6 markedly reduced the EMT reprogramming, the cell migration, and invasion in active PLK1-driven or TGF-β-induced EMT, indicating that TSG6 would be a critical factor in PLK1-mediated metastasis. Although treatment of TGF-β or active PLK1 upregulates TSG6 expression, the depletion of TSG6 reduced the levels of *PLK1* or *TGFB1*, suggesting that TSG6 and PLK1 or *TGFB1* could mutually trigger their expression for cancer metastasis (Fig. 6k). Thus, active PLK1-driven metastasis is amplified by TGF-β signaling and TSG6, which forms a positive feedback loop.

For colonization after the EMT, cells have to undergo MET for tumorigenic progression. The difficulties of metastasis are changing the cell traits. Interestingly, many studies have reported that some metastatic cancers are grouped with different traits [7, 11, 12]. In our studies of functional domains, the expression of the phosphomimetic p-S137-PLK1 mutant produced epithelial characters with high levels of *CDH1*. Meanwhile, p-T210-PLK1 showed both motility for migration to distant regions and tumorigenicity for colonization. Because of the differential effects of phosphorylation residues in PLK1, the overexpression of PLK1 in cancer could promote migration, invasion, and colonization, through the differential phosphorylation status. Of note, the differential phosphorylation can regulate the metastasis status. Until now, it has been unclear how motility and colonization could be regulated for metastasis because the epithelial and mesenchymal characters differ. Based on this study, we propose the possibility that the differential phosphorylation status of critical factors in tumorigenesis and/or metastasis might modulate the characteristics of cancer between epithelial and mesenchymal status for metastasis and colonization in the second spot.

In conclusion, active PLK1 phosphorylated at T210, abundant in TGF-β-induced metastatic NSCLC, promotes metastasis through the upregulation of genes related to the TGF-β-related signaling pathway including *TNFAIP6*, which amplifies NSCLC’s metastatic properties by forming a positive loop. The loss of *TNFAIP6*, top-ranked gene highly expressed in invasive cells expressing active PLK1, disturbed the metastasis induced by active PLK1 or TGF-β. Clinical data provide evidence for the connection between the expression of *PLK1* and *TNFAIP6* and the survival rates of NSCLC patients. Combining *PLK1* with *TNFAIP6* has a more significant prognostic association than either gene alone in metastatic NSCLC, suggesting that high expression of both *TNFAIP6* and *PLK1* could be a negative prognostic marker for survival among metastatic NSCLC patients and that PLK1 and TSG6 are valuable therapeutic targets for metastatic NSCLC treatment.

## Materials and Methods

### Materials

Human lung cells from the MRC5, A549, NCI-H1299, NCI-H358, and NCI-H460 cell lines were purchased from KCLB (KCLB; Seoul, Korea). HEK293T cells were purchased from ATCC (ATCC; Manassas, VA, USA). Minimum essential medium Eagle (MEM), RPMI 1640 medium, Dulbecco’s modified Eagle’s medium (DMEM), fetal bovine serum (FBS), penicillin, and streptomycin were purchased from Corning (Detroit, MI, USA). Transforming growth factor (TGF)-β and all other chemical reagents were purchased from Sigma-Aldrich (St. Louis, MO, USA).

### Cell culture and treatment

MRC5 and A549 cells were cultured in MEM (Corning), NCI-H1299 and NCI-H460 cells in RPMI 1640 medium (Corning), and HEK293T cells (ATCC) in DMEM (Corning). These cells were supplemented with 10% FBS in the presence of antibiotics in a humidified 5% CO_2_ incubator at 37°C. For the TGF-β treatment, cells were seeded at 1 × 10^4^ cells/ml, and after 16 h, the cells were treated with 2.5 ng/ml TGF-β for 48 h.

### Lentivirus-based plasmid preparation, virus production, and infection

For lentiviral expression of mouse PLK1 (gene ID no. 18817), we used the pLVX-TRE3G-eRFP and pLVX-Tet3G vectors (Clontech #631351; Palo Alto, CA, USA). Wild type, S137D, T210D, S137D/T210D, K82M, and W414F/V415A mutant PLK1 were described previously [23]. The wild type and various versions of PLK1 were amplified using a forward primer (5′-ACGGGGCCCATGAGTGCTGCAGTGA-3′) and a reverse primer (5′- ACGACGCGTTTAGGAGGCCTTGAGA-3′) and subcloned into pLVX-TRE3G-eRFP using the *Apa*I and *Mlu*I restriction enzymes according to the manufacturer’s guide and a previous report [40]. Lentivirus expressing PLK1 was generated according to the manufacturer’s protocol. Briefly, pLVX-TRE3G-eRFP-PLK1 or pLVX-Tet3G was transfected into HEK293T cells with the pCMV-delta R8.2 packaging plasmid and the pCMV-VSV.G envelope plasmid. Medium was replaced after 24 h, and the supernatant collected 36, 48, 60, and 72 h after transfection. For viral infection, cells were treated with the viral particles from pLVX-Tet3G and pLVX-TRE3G-eRFP-PLK1 in the presence of 10 µg/ml polybrene and 10 mM HEPES. The infected cells were selected using 500 µg/ml G418 for 5 days and 2 µg/ml puromycin for 3 days. The expression of PLK1 was induced by treatment with 1 µg/ml doxycycline.

### Lentivirus-based shRNA preparation

For the loss of function experiments, we prepared lentivirus-based shRNA transfer plasmids targeting human PLK1 (gene access no. NM_005030) at positions 1424–1444 (AGATCACCCTCCTTAAATATT) (pLKO-Puro.1-PLK1#1) or at 245–265 (AGATTGTGCCTAAGTCTCTGC) (pLKO-Puro.1-PLK#2), and human TNFAIP6 (gene access no. NM_007115) at positions 693–713 (GGGAAGATACTGTGGAGATGA) (pLKO-Puro.1-TNFAIP6), and then the lentivirus was generated as described previously [41, 42]. The infected cells were selected using 2 µg/ml puromycin for 3 days.

### Immunoblot analysis

Cells were lysed in lysis buffer [0.5% Triton X-100, 20 mM Tris (pH 7.5), 2 mM MgCl_2_, 1 mM dithiothreitol, 1 mM EGTA, 50 mM β-glycerophosphate, 25 mM NaF, 1 mM Na vanadate, 100 mg/ml phenylmethanesulfonyl fluoride (PMSF), and protease inhibitor cocktail (Roche; Indianapolis, IN, USA)]. After adjusting the protein concentration, proteins were resolved by sodium dodecyl sulfate–polyacrylamide gel electrophoresis (SDS–PAGE) and subjected to immunoblot analysis with the indicated antibodies as follows: PLK1 (Millipore, 05–844); phospho-PLK1 T210 (Cell Signaling, 5472); phospho-PLK1 S137 (Abcam, ab21738); Vimentin (Santa Cruz Biotechnology, sc7557); E-cadherin (Cell Signaling, 4065); N-cadherin (Sigma, C3865); snail (Santa Cruz Biotechnology, sc271977); slug (Cell Signaling, 9585); GAPDH (Sigma, G8795); phospho-smad2 S465/S467 (Cell Signaling, 18338); Smad2/3 (Cell Signaling, 8685); Ki-67 (Abcam, ab16667); β-actin (Sigma, A5441); and RFP (Life Technologies, R10367). Immune complexes were displayed using an Odyssey infrared imaging system (LI-COR Biosciences; Lincoln, NE, USA) (Supplementary Fig. S8). Intensity values were determined using LI-COR Odyssey software.

### Cell wound-healing assay

For the cell motility assay, A549 or NCI-H460 cells were plated at 1 × 10^5^ cells/well in 6-well plates, and a wound was established by scratching one time with a 1 mm thick pipette tip. The detached cells were removed by washing with phosphate-buffered saline (PBS). Migration of cells into the wounded region was observed, and the images were analyzed with an Eclipse Ti microscope (Nikon; Tokyo, Japan) at the indicated times. The wound area was measured using Nikon NIS-Elements image processing software (Nikon; Tokyo, Japan).

### Transwell cell-migration and invasion assay

Cell migration assays were conducted using 24-well plates with 8-μm pore Transwell chambers (Corning, NY, USA). The lower chamber was filled with culture medium containing 10% FBS. A549 or NCI-H460 cells were suspended at a density of 5 × 10^4^ cells in MEM or 1 × 10^5^ cells in RPMI medium, respectively, without FBS and added to the upper chamber. Three days after seeding, the cells on the bottom layer surface were stained with 0.05% crystal violet dye, and the intensity values were measured using an Odyssey infrared imaging system (LI-COR Biosciences). For the cell invasion assay, cells were seeded in the upper chamber filled with Matrigel (BD Biosciences, Erembodegem, Belgium). Five to 7 days after seeding, the cells on the bottom layer surface were stained with 0.05% crystal violet dye, and after treatment with dimethyl sulfoxide (DMSO), the absorbance was measured at 590 nm using an M4 microplate reader (Molecular Devices, CA, USA).

### Colony formation assay

In all, 5 × 10^3^ cells were resuspended in 2 ml of medium with 10% FBS in 0.4% agar and overlaid onto the bottom agar layer composed of 10% FBS and 0.6% agar in 1.5 ml of medium in a 35-mm dish. The cells were incubated in a humidified 5% CO_2_ incubator at 37°C. After 4 weeks, the colonies formed in the agar were counted after staining with 0.05% crystal violet.

### Quantitative real-time polymerase chain reaction (qRT-PCR)

Total RNA was extracted 48 h after exposure to TGF-β and quantified by Nanodrop (Thermo Scientific; Wilmington, DE, USA). Complementary DNA (cDNA) was synthesized with a First Strand cDNA Synthesis Kit (Thermo Scientific) and then mixed with SYBR Green Master Mix (Roche; Mannheim, Germany) and gene-specific primers. qRT-PCR was performed using a LightCycler Real-Time PCR system (Roche). The sequences of primers used for the qRT-PCR are in Supplementary Table S3.

### Experimental lung metastasis assay

Four-week-old male BALB/c nude mice (Orient Bio, Seoul, Korea) were injected with A549 cells stably expressing pLVX-TRE3G-eRFP-Tet3G-Mock, WT-PLK1, T210D-PLK1, or W414F/V415A-PLK1 (2 × 10^6^ cells/100 μl PBS) via the tail vein. The mice received 1 mg/ml of doxycycline in their drinking water to induce TRE3G-PLK1 overexpression. Ten weeks after injection, all mice were sacrificed, and their lungs were separated and fixed in 4% paraformaldehyde for H&E tissue staining. To observe the effects of the PLK1 inhibitor volasertib on metastasis, cells expressing T210D-PLK1 were injected (2 × 10^6^ cells/100 μl PBS) through the tail vein of 4-week-old male BALB/c nude mice. Two weeks after injection, the mice were treated with 20 mg/kg volasertib (0.1 N HCl, diluted in PBS) once a week via tail-vein injection for 3 weeks. All animal experiments were approved and managed by the guidelines of the Institutional Animal Care and Use Committee, Hanyang University (HY-IACUC-2017-0115A).

### Bioinformatics analysis

NSCLC patients’ data were obtained from the online database (https://software.broadinstitute.org/morpheus) and (www.kmplot.com) according to the previous report [26]. All cancer patients in the database were identified from the Cancer Biomedical Informatics Grid (caBIG, http://cabig.cancer.gov/, microarray samples are published by the caArray project), the Gene Expression Omnibus (GEO, http://www.ncbi.nlm.nih.gov/geo/) and the Cancer Genome Atlas (TCGA, http://cancergenome.nih.gov) cancer datasets. The database, which was established using gene expression data and survival information about 1926 NSCLC patients, was used to establish the clinical relevance of PLK1 expression to the survival rates of NSCLC patients after excluding biased arrays. The expression values for PLK1 and clinical data for those samples were extracted and used for the survival analysis. The samples were split into high and low groups using PLK1 expression. Hazard ratios (HRs) with 95% confidence intervals and the log rank *p* were calculated according formulas on the webpage. A *p*-value of <0.05 was considered to be statistically significant. An HR is the ratio of the hazard rates corresponding to the conditions described by two levels of an explanatory variable in a survival analysis.

### Transcriptome profiling

RNA was extracted from the indicated invasive cells and non-invasive cells expressing mock, wild type, or constitutively active PLK1 (T210D). RNA purity and integrity were evaluated using an ND-1000 spectrophotometer (NanoDrop, Wilmington, USA) and Agilent 2100 bioanalyzer (Agilent Technologies, Palo Alto, USA). The Affymetrix whole-transcript expression array process was executed according to the manufacturer’s protocol (GeneChip Whole Transcript PLUS reagent kit). cDNA was synthesized using the GeneChip WT (Whole Transcript) Amplification kit as described by the manufacturer. The sense cDNA was then fragmented and biotin-labeled with terminal deoxynucleotidyl transferase using the GeneChip WT Terminal labeling kit. Approximately 5.5 μg of labeled DNA target was hybridized to the Affymetrix GeneChip Human 2.0 ST Array at 45 °C for 16 h. Hybridized arrays were washed and stained on a GeneChip Fluidics Station 450 and scanned on a GCS3000 Scanner (Affymetrix). Signal values were computed using the Affymetrix® GeneChip™ Command Console software.

### Microarray analysis

Raw data were extracted automatically using an Affymetrix data extraction protocol in the Affymetrix GeneChip® Command Console® software. After importing CEL files, the data were summarized and normalized with the robust multi-average (RMA) method implemented in the Affymetrix® Expression Console™ software. We exported the results of the gene level RMA analysis and performed a differentially expressed gene analysis. The analysis comparing the wild type PLK1 or constitutive active PLK1 with the non-invasive mock was carried out using fold changes. For transcriptome data, gene probes with significant fold changes (>1.5) were clustered. To develop a significant probe list, we performed a gene-enrichment and functional annotation analysis using gene ontology (http://geneontology.org/) and KEGG (http://kegg.jp). All statistical tests and visualizations of differentially expressed genes were conducted using R statistical language v. 3.1.2. (www.r-project.org).

### Statistical analysis

All data are given as the means ± SDs of at least three independent experiments, each performed in triplicate. Results were analyzed for statistically significant differences using the student’s *t*-test, and statistical significance was set at *p* < 0.05 (*p *<* 0.05; **p *<* 0.01; ***p *<* 0.001).

## Supplementary information

Supplementary Information

Supplemental Table S1

Supplemental Table S2

Supplemental Table S3

Supplementary Figure S1

Supplementary Figure S2

Supplementary Figure S3

Supplementary Figure S4

Supplementary Figure S5

Supplementary Figure S6

Supplementary Figure S7

Supplementary Figure S8

## Data Availability

The authors declare that all the data supporting the findings of this study are available within the paper and its supplementary information files. All other data supporting the findings of this study are available from the corresponding authors upon reasonable request.
