# Supplementary material for: Active PLK1-driven metastasis is amplified by TGF-β signaling that forms a positive feedback loop in non-small cell lung cancer
Source: Oncogene. 2019 Sep 23;39(4):767–85. doi: 10.1038/s41388-019-1023-z (PMC6976524; doi:10.1038/s41388-019-1023-z)
Supplement: Supplementary file 1 — Supplementary Information [file 41388_2019_1023_MOESM1_ESM.doc]

**Supplementary Information**

**Active PLK1-driven metastasis is amplified by TGF-β signaling that forms a positive feedback loop in non-small cell lung cancer**

Sol-Bi Shin^†^, Hay-Ran Jang^†^, Rong Xu^†^, Jae-Yeon Won^†^, and Hyungshin Yim^*†^

^†^Department of Pharmacy, College of Pharmacy, **Institute of Pharmaceutical Science and Technology,** Hanyang University, Ansan, Gyeonggi-do, Korea

^*^Corresponding author

Hyungshin YIM

Address: Department of Pharmacy, College of Pharmacy, **Institute of Pharmaceutical Science and Technology,** Hanyang University, Ansan, Gyeonggi-do 15588, Korea

Phone: +82-31-400-5810

FAX: +82-31-400-5958

E-mail: [hsyim@hanyang.ac.kr](mailto:hsyim@hanyang.ac.kr)

**Running title**: Amplification of PLK1-driven metastasis by TGF-β signaling

**Potential conflicts of interest**

The authors declare that there is no potential conflicts of interest.

**Supplementary Figure Legends**

**Supplementary Figure S1. Genomic analyses of PLK1, epithelial markers, mesenchymal markers, and proliferation factors in NSCLC patients.** Heat maps were generated from TCGA lung squamous cell carcinoma patients’ dataset. Heat map showed the expression profile of genes including PLK1, epithelial markers (Epi), mesenchymal markers (Mes), and proliferation markers (Pro) in paired normal and tumor tissues with stage 1 (right) or stages 2-4 (left).

**Supplementary Figure S2. A scheme of the functional domain and mutagenesis residue of PLK1. (A)** The structures of wild type PLK1 (WT), a constitutively active phosphomimic form at T210 (TD), a phosphomimic form at S137 (SD), a double phosphomimic form at S137 and T210 (SDTD), a kinase-defective form (KM), and a form with a mutation in the polo-box domain (FA) are displayed. **(B)** A549 cells expressing wild type PLK1, active T210D mutant, kinase-defective K82M PLK1 were subjected to immunoblotting using specific antibodies against for TCTP, p-TCTP, PLK1, and β-actin (upper panel). The relative intensity of pTCTP/TCTP was measured and plotted (lower panel).

**Supplementary Figure S3. Expression of catalytically active p-T210-PLK1 mimic mutant induces migration.** A549 **(A)** and NCI-H460 **(B)** cells expressing various versions of PLK1 were subjected to a wound healing assay for 72 hours. TGF-β was used as a positive control. The scratch recovery efficiency after 72 hours was analyzed using NIS-Elements Imaging software (Nikon, Japan).

**Supplementary Figure S4. Depletion of PLK1 suppresses PLK1-driven migration. (A)** A549 cells were infected by lentiviral PLK1 shRNA #1 or #2 and cell proliferation assay was performed. **(B)** A549 cells were infected by lentiviral PLK1 shRNA #1 or #2 and treated TGF-β for 48 hours and cell migration assay was performed. **p* <0.05; ***p* <0.01; ****p* <0.001 (*n*=3) Data presented as mean ± SEM. **(C)** After A549 cells were treated by poloxin or volasertib and cell proliferation assay was performed. **(D-E)** A549 cells expressing eRFP-tagged TD were treated with shRNA targeting human PLK1 **(D)** or volasertib **(E),** and an *in vitro* wound-healing assay was performed. The scratch recovery efficiency was analyzed 24, 48, and 72 hours after scratching using NIS-Elements Imaging software (Nikon, Japan). The relative migration distance was measured and plotted (right).

**Supplementary Figure S5. Scheme of experiments.**

**Supplementary Figure S6. Relative gene expression profile of invasive A549 cells expressing TD.** Analysis of transcriptome data for gene probes in the invasive cells expressing TD, compared with those of non-invasive A549 cells expressing TD.

**Supplementary Figure S7. Cumulative overall survival of all NSCLC patients and TMN stage N2 patients according to gene expression.** **(A)** Cumulative overall survival of all NSCLC patients (left) and TMN stage N2 patients (right) according to the expression of *TNFAIP6* or *PLK1* gene alone. **(B)** Cumulative overall survival of all NSCLC patients (left) and TMN stage N2 patients (right) according to *TNFAIP6* expression.

**Supplementary Figure S8. Full-length immunoblotting images.**

**Supplementary Table Legends**

**Supplemental Table S1.** The list of genes differentially expressed in cells expressing wild type or active T210D PLK1.

**Supplemental Table S2.** Downregulated genes targeted by MIR3167 in cells expressing wild type or active T210D PLK1.

**Supplemental Table S3.** Sequences of forward (F) and reverse (R) primers used for RT-PCR amplification.
