# Supplementary material for: Active PLK1-driven metastasis is amplified by TGF-β signaling that forms a positive feedback loop in non-small cell lung cancer
Source: Oncogene. 2019 Sep 23;39(4):767–85. doi: 10.1038/s41388-019-1023-z (PMC6976524; doi:10.1038/s41388-019-1023-z)
Supplement: Supplementary file 2 — Supplemental Table S1 [file 41388_2019_1023_MOESM2_ESM.doc]

**Supplementary Table S1. The list of genes differentially expressed** in cells expressing wild type or active T210D PLK1.

| **Gene name** | **Gene Access No.** | **Noninvasive WT** | **Noninvasive TD** | **Invasive WT** | **Invasive TD** |
| --- | --- | --- | --- | --- | --- |
| LCE3D | NM_032563 | 1.26 | **-2.15** | **1.581** | **7.780** |
| TNFAIP6 | NM_007115 | -1.08 | **-2.96** | 1.152 | **6.618** |
| LAMC2 | NM_005562 | -1.11 | **-3.51** | 1.131 | **4.197** |
| MIR3167 | NR_036126 | 1.26 | **-1.78** | 1.102 | **3.927** |
| RASGRP3 | NM_001139488 | -1.26 | **-2.36** | 1.047 | **3.886** |
| IGFL3 | NM_207393 | -1.04 | **-1.90** | 1.312 | **3.849** |
| IGFL1 | NM_198541 | 1.05 | **-2.55** | -1.370 | **3.819** |
| LOC105374715 | XR_925901 | 1.09 | **-2.06** | -1.050 | **3.809** |
| MARCH4 | NM_020814 | 1.08 | **-1.95** | **1.746** | **3.807** |
| LOC105369559 | XR_913665 | 1.07 | **-1.79** | 1.176 | **3.806** |
| TSPAN2 | NM_001308315 | -1.14 | **-2.04** | 1.001 | **3.785** |
| ITGA11 | NM_001004439 | 1.02 | **-1.96** | 1.028 | **3.464** |
| NPTX1 | NM_002522 | 1.41 | **-1.76** | 1.044 | **3.149** |
| IFNE | NM_176891 | 1.10 | -1.47 | -1.217 | **3.142** |
| SNORD114-17 | NR_003210 | **2.36** | -1.03 | **2.243** | **3.112** |
| GXYLT2 | NM_001080393 | 1.13 | **-1.50** | **1.654** | **3.065** |
| RASGRF2 | NM_006909 | 1.29 | **-1.71** | 1.111 | **2.902** |
| IL11 | NM_000641 | -1.01 | **-2.31** | -1.225 | **2.900** |
| ADAM19 | NM_033274 | -1.03 | **-2.26** | 1.015 | **2.847** |
| LOC105369301 | XR_920493 | -1.01 | **-1.72** | 1.174 | **2.796** |
| LOC105376382 | XR_930613 | -1.32 | **-2.21** | -1.038 | **2.703** |
| SNORD113-1 | NR_003229 | 1.30 | -1.38 | 1.119 | **2.682** |
| SNHG24 | NR_110178 | 1.33 | -1.06 | **2.213** | **2.674** |
| SLN | NM_003063 | -1.24 | **-1.89** | 1.005 | **2.617** |
| COL4A1 | NM_001303110 | 1.13 | **-1.87** | 1.111 | **2.600** |
| MIR181B2 | NR_029782 | 1.14 | -1.35 | 1.174 | **2.585** |
| SNORD113-4 | NR_003232 | **1.51** | -1.27 | **1.560** | **2.573** |
| SNORD114-3 | NR_003195 | 1.43 | -1.18 | **3.111** | **2.563** |
| MMP2 | NM_001127891 | 1.01 | **-2.14** | 1.113 | **2.494** |
| CCDC80 | NM_199511 | **-1.94** | **-3.47** | **-1.620** | **2.489** |
| R3HDML | NM_178491 | 1.12 | **-1.53** | -1.031 | **2.460** |
| LOC105372424 | XR_919673 | -1.43 | **-2.23** | -1.078 | **2.451** |
| EVA1A | NM_001135032 | 1.46 | -1.47 | 1.285 | **2.405** |
| CEP295NL | ENST00000586713 | -1.09 | -1.28 | 1.222 | **2.373** |
| TPM1 | NM_000366 | -1.20 | **-1.84** | -1.031 | **2.365** |
| C15orf48 | NM_032413 | 1.03 | -1.37 | 1.462 | **2.358** |
| CACHD1 | NM_001293274 | 1.15 | -1.39 | 1.391 | **2.340** |
| SPOCK1 | NM_004598 | -1.05 | **-1.90** | 1.025 | **2.316** |
| THBS1 | NM_003246 | -1.15 | **-2.46** | -1.186 | **2.313** |
| LOC105371809 | XR_918455 | -1.29 | **-2.37** | -1.180 | **2.304** |
| CD44 | AF086543 | **1.55** | 1.23 | **1.727** | **2.291** |
| LOC101929475 | XR_242755 | -1.17 | **-1.80** | 1.473 | **2.287** |
| LINC00342 | AK057596 | 1.04 | **-2.02** | -1.208 | **2.276** |
| LOC400212 | XR_915606 | -1.13 | -1.41 | 1.158 | **2.266** |
| SERPINE1 | NM_000602 | **-1.55** | **-4.20** | **-1.826** | **2.245** |
| LCE3E | NM_178435 | 1.07 | -1.45 | 1.001 | **2.241** |
| MIR548C | NR_030347 | -1.08 | -1.01 | **1.652** | **2.240** |
| HNRNPA1P33 | NR_003277 | -1.34 | **-2.43** | -1.001 | **2.232** |
| KCNMA1 | NM_001014797 | -1.09 | **-2.51** | -1.078 | **2.219** |
| COL4A2 | NM_001846 | -1.05 | **-1.73** | 1.096 | **2.219** |
| LOC105374715 | XR_925901 | 1.13 | -1.39 | 1.320 | **2.191** |
| SNORD116-30 | NR_047032 | **2.08** | 1.20 | **2.018** | **2.184** |
| MEG3 | NR_002766 | **1.74** | -1.24 | 1.091 | **2.167** |
| FOXP1-IT1 | AK025793 | **1.58** | -1.01 | 1.090 | **2.160** |
| KIAA1549L | NM_012194 | 1.09 | **-1.82** | 1.058 | **2.156** |
| IGFBP7 | NM_001253835 | 1.02 | **-1.92** | 1.041 | **2.137** |
| KLHL5 | XM_011513700 | -1.02 | -1.11 | **1.611** | **2.137** |
| SNORD114-28 | NR_003221 | 1.43 | 1.10 | **2.259** | **2.126** |
| SNORD114-10 | NR_003203 | 1.43 | 1.17 | **1.910** | **2.111** |
| MIR181A2HG | NR_038975 | 1.23 | -1.16 | 1.184 | **2.095** |
| LINC00842 | NR_033957 | **-1.69** | **-2.35** | -1.064 | **2.093** |
| PGM2L1 | NM_173582 | -1.07 | -1.34 | -1.030 | **2.077** |
| SERPINE2 | NM_001136528 | -1.09 | **-1.79** | 1.013 | **2.071** |
| STC1 | NM_003155 | 1.42 | 1.44 | **2.119** | **2.063** |
| SLC16A2 | NM_006517 | 1.31 | **-1.62** | 1.459 | **2.050** |
| LOC105376439 | XR_930718 | 1.26 | -1.13 | 1.343 | **2.049** |
| SULF2 | NM_001161841 | 1.10 | **-1.63** | 1.191 | **2.035** |
| GALNT18 | NM_198516 | 1.10 | **-1.67** | 1.074 | **2.030** |
| LOC105373161 | XR_921289 | **2.29** | 1.24 | 1.458 | **2.022** |
| P4HA3 | NM_001288748 | -1.37 | **-1.67** | -1.202 | **2.010** |
| KCNJ6 | NM_002240 | 1.14 | -1.28 | 1.183 | **2.006** |
| MIR582 | NR_030308 | **1.55** | -1.09 | 1.119 | **2.005** |
| KCNH1 | NM_002238 | -1.14 | **-1.93** | -1.015 | **1.990** |
| MIR31HG | NR_027054 | -1.33 | -1.25 | 1.082 | **1.988** |
| GABRQ | NM_018558 | **-2.29** | **-3.19** | **-1.555** | **1.980** |
| CMTM3 | NM_144601 | 1.15 | **-1.54** | 1.126 | **1.979** |
| CLMP | NM_024769 | 1.21 | **-1.86** | 1.477 | **1.963** |
| TIMP3 | NM_000362 | 1.20 | -1.19 | 1.356 | **1.955** |
| PMEPA1 | NM_001255976 | -1.25 | **-1.78** | -1.208 | **1.950** |
| FLRT2 | NM_013231 | 1.13 | -1.36 | 1.128 | **1.935** |
| LOC105374003 | XR_924265 | 1.14 | -1.21 | **1.591** | **1.934** |
| VGLL3 | NM_016206 | 1.20 | -1.17 | 1.417 | **1.931** |
| LRRC8C | NM_032270 | -1.23 | **-1.92** | -1.168 | **1.930** |
| MIR181A2 | NR_029611 | 1.20 | -1.20 | -1.045 | **1.930** |
| DPYSL3 | NM_001197294 | 1.08 | **-1.57** | **1.515** | **1.926** |
| ITGB6 | NM_000888 | **-2.59** | **-3.71** | **-2.404** | **1.924** |
| HMGN2P46 | NR_022014 | 1.23 | -1.15 | 1.130 | **1.921** |
| FSTL1 | NM_007085 | -1.03 | **-1.86** | 1.191 | **1.918** |
| PLEK2 | NM_016445 | **-1.75** | **-2.71** | -1.142 | **1.917** |
| SNORD114-6 | NR_003198 | 1.08 | **-1.68** | **-1.531** | **1.909** |
| MIR1206 | NR_031611 | -1.39 | 1.40 | 1.493 | **1.908** |
| ROS1 | NM_002944 | -1.03 | **-1.71** | -1.392 | **1.899** |
| LINC01474 | NR_121120 | 1.15 | 1.31 | 1.105 | **1.894** |
| IGKV1OR2-108 | OTTHUMT00000330788 | 1.02 | -1.10 | 1.241 | **1.890** |
| SLIT3 | NM_001271946 | 1.24 | -1.37 | 1.282 | **1.885** |
| TNS1 | NM_001308022 | 1.11 | -1.38 | -1.143 | **1.882** |
| CGB8 | NM_033183 | **1.96** | -1.12 | 1.132 | **1.882** |
| DSC2 | NM_004949 | -1.09 | -1.41 | 1.209 | **1.878** |
| NIPAL4 | NM_001099287 | 1.42 | -1.36 | 1.260 | **1.877** |
| PIK3AP1 | NM_152309 | 1.08 | -1.29 | 1.375 | **1.870** |
| CYR61 | NM_001554 | 1.16 | -1.07 | **1.663** | **1.868** |
| LOC729732 | NR_047662 | 1.33 | -1.01 | **1.927** | **1.868** |
| SNORD113-3 | NR_003231 | -1.13 | -1.13 | **1.715** | **1.864** |
| TPST1 | NM_003596 | -1.07 | -1.49 | 1.124 | **1.861** |
| FGFR1 | NM_001174063 | 1.09 | -1.28 | 1.290 | **1.858** |
| LTBP4 | NM_001042544 | 1.13 | -1.39 | 1.166 | **1.857** |
| PDLIM7 | NM_005451 | -1.10 | **-1.93** | -1.221 | **1.856** |
| MIR4714 | NR_039864 | 1.10 | 1.24 | 1.480 | **1.855** |
| IGANRP | AB586692 | 1.01 | -1.27 | -1.170 | **1.849** |
| KCNJ15 | NM_001276435 | -1.06 | -1.38 | 1.076 | **1.847** |
| MIR554 | NR_030280 | -1.10 | -1.36 | 1.015 | **1.845** |
| NRG1 | NM_001159995 | -1.13 | **-1.59** | -1.032 | **1.844** |
| LOC645513 | ENST00000498873 | 1.19 | -1.37 | 1.116 | **1.843** |
| FST | NM_006350 | 1.02 | -1.39 | -1.066 | **1.840** |
| MIR1284 | NR_031697 | -1.21 | **-1.53** | -1.105 | **1.839** |
| MIR3160-1 | NR_027334 | **2.05** | 1.03 | 1.246 | **1.838** |
| EPHB2 | NM_001309192 | -1.39 | **-2.73** | -1.356 | **1.838** |
| MIR548B | NR_030315 | 1.44 | 1.14 | **1.902** | **1.831** |
| ITGA5 | NM_002205 | 1.33 | -1.12 | **1.580** | **1.826** |
| GALNT16 | NM_001168368 | 1.18 | -1.39 | **1.671** | **1.819** |
| DOCK4 | NM_014705 | 1.06 | -1.11 | **1.506** | **1.814** |
| LINC00862 | NR_040064 | **-1.50** | **-1.93** | -1.079 | **1.813** |
| ANXA8L1 | OTTHUMT00000047846 | 1.07 | **-1.53** | -1.031 | **1.810** |
| TGFBI | NM_000358 | -1.01 | **-1.55** | -1.173 | **1.810** |
| TMEM88 | NM_203411 | 1.20 | -1.39 | -1.170 | **1.807** |
| IGFL2 | NM_001002915 | 1.19 | -1.31 | -1.293 | **1.803** |
| IFI6 | NM_002038 | 1.25 | -1.22 | 1.441 | **1.803** |
| CORO2B | NM_001190456 | -1.05 | **-1.61** | -1.061 | **1.800** |
| PCDHB6 | NM_001303145 | 1.32 | 1.03 | **2.947** | **1.797** |
| LOC105376603 | XR_931144 | 1.32 | -1.10 | 1.340 | **1.797** |
| ADGRF2 | NM_153839 | -1.03 | -1.33 | -1.128 | **1.795** |
| LOC105371809 | XR_918455 | -1.26 | **-1.69** | -1.095 | **1.792** |
| RTKN2 | NM_001282941 | -1.06 | **-1.63** | -1.195 | **1.792** |
| TGFB1I1 | NM_001042454 | **-1.71** | **-3.13** | **-1.597** | **1.784** |
| RASGRP1 | NM_001128602 | 1.03 | **-1.58** | -1.184 | **1.780** |
| MAFK | NM_002360 | 1.37 | 1.27 | **1.532** | **1.779** |
| BEND6 | NM_003770 | 1.39 | 1.06 | 1.218 | **1.779** |
| CCBE1 | NM_133459 | 1.19 | -1.33 | 1.277 | **1.775** |
| FERMT1 | NM_017671 | 1.15 | -1.19 | 1.395 | **1.774** |
| TGFBR1 | NM_001130916 | -1.42 | **-1.95** | -1.350 | **1.759** |
| NT5E | NM_001204813 | 1.01 | -1.36 | 1.058 | **1.754** |
| TUFT1 | NM_001126337 | -1.05 | **-1.56** | 1.009 | **1.754** |
| LOC100130502 | XR_244997 | **1.63** | -1.02 | 1.289 | **1.752** |
| DOCK2 | NM_004946 | -1.38 | **-1.87** | -1.086 | **1.752** |
| F2RL1 | NM_001003674 | 1.26 | -1.26 | 1.422 | **1.751** |
| SLC2A3 | NM_006931 | **1.50** | 1.28 | 1.419 | **1.750** |
| ANGPTL4 | NM_001039667 | 1.31 | -1.05 | **1.544** | **1.749** |
| SNORD99 | NR_003077 | **1.55** | 1.26 | **1.681** | **1.748** |
| ARHGAP11B | OTTHUMT00000430733 | -1.33 | -1.18 | **1.582** | **1.748** |
| BEAN1 | NM_001136106 | 1.22 | -1.41 | 1.168 | **1.745** |
| SLFNL1-AS1 | NR_037868 | 1.38 | -1.14 | 1.151 | **1.741** |
| RNF182 | NM_001165032 | 1.24 | -1.05 | **1.565** | **1.740** |
| CASC10 | NM_001010911 | -1.01 | -1.11 | **1.671** | **1.737** |
| SNAI2 | NM_003068 | 1.08 | -1.43 | 1.165 | **1.735** |
| PACS1 | NM_018026 | -1.01 | -1.23 | 1.076 | **1.735** |
| DDB2 | NM_000107 | 1.05 | **-1.79** | -1.038 | **1.732** |
| MIR3192 | NR_036160 | **1.57** | **1.58** | **1.996** | **1.726** |
| LOC105373206 | XR_921375 | 1.47 | 1.00 | 1.402 | **1.726** |
| EFR3B | NM_014971 | -1.34 | **-1.90** | 1.079 | **1.726** |
| CMIP | ENST00000566462 | 1.13 | -1.05 | -1.115 | **1.724** |
| EGOT | NR_004428 | -1.27 | **-1.64** | -1.066 | **1.722** |
| SNRPN | AF319524 | 1.14 | -1.03 | -1.131 | **1.721** |
| SNORD114-20 | NR_003213 | 1.37 | -1.03 | 1.352 | **1.721** |
| MBOAT2 | NM_138799 | **-1.63** | **-1.94** | -1.008 | **1.720** |
| LOC100131541 | AY358248 | 1.06 | -1.17 | -1.028 | **1.715** |
| FHOD3 | NM_001281739 | -1.09 | **-1.65** | -1.033 | **1.711** |
| FRMD6 | NM_001042481 | -1.12 | **-1.52** | 1.040 | **1.708** |
| INPP4B | NM_001101669 | -1.26 | **-1.51** | -1.143 | **1.708** |
| GOLGA8J | NM_001282472 | -1.05 | -1.02 | 1.485 | **1.706** |
| TIMP2 | NM_003255 | -1.16 | **-1.79** | -1.023 | **1.702** |
| MIR520E | NR_030183 | **1.61** | 1.46 | 1.439 | **1.702** |
| FAM49A | NM_030797 | 1.06 | **-1.57** | -1.103 | **1.701** |
| LOC100507639 | NR_121625 | 1.16 | -1.10 | -1.254 | **1.700** |
| KDM6B | NM_001080424 | 1.00 | -1.44 | -1.058 | **1.695** |
| TKTL1 | NM_001145933 | **1.70** | 1.19 | 1.315 | **1.695** |
| LINC00322 | AK131425 | 1.18 | -1.05 | 1.080 | **1.691** |
| SEMA3C | NM_006379 | 1.08 | -1.39 | 1.013 | **1.688** |
| ADGRF4 | NM_153838 | -1.08 | **-1.59** | -1.171 | **1.687** |
| LOC105375675 | XR_928460 | **1.75** | 1.14 | 1.480 | **1.685** |
| F3 | NM_001178096 | -1.04 | **-1.60** | -1.311 | **1.683** |
| TNFRSF12A | NM_016639 | 1.00 | **-1.88** | 1.007 | **1.681** |
| C11orf80 | NM_001302084 | -1.32 | **-1.59** | -1.199 | **1.681** |
| MIR924 | NR_030628 | **1.67** | 1.11 | 1.342 | **1.680** |
| MT1X | NM_005952 | 1.10 | **-1.55** | -1.289 | **1.679** |
| TP53I3 | NM_001206802 | -1.49 | **-2.05** | -1.204 | **1.678** |
| IL32 | NM_001012631 | 1.26 | -1.17 | 1.388 | **1.678** |
| SNORD116-20 | NR_003334 | 1.45 | -1.03 | **2.045** | **1.677** |
| FIBCD1 | NM_001145106 | 1.32 | -1.25 | 1.016 | **1.677** |
| CNIH2 | NM_182553 | 1.37 | 1.15 | 1.336 | **1.676** |
| LOC645166 | NR_027354 | 1.26 | -1.16 | 1.148 | **1.674** |
| F2R | NM_001311313 | 1.30 | -1.19 | **1.563** | **1.671** |
| ADAMTS6 | NM_197941 | 1.06 | -1.21 | 1.130 | **1.670** |
| ZNF680 | NM_001130022 | -1.29 | -1.39 | 1.351 | **1.668** |
| IER3 | NM_003897 | 1.07 | -1.16 | 1.264 | **1.668** |
| JUNB | NM_002229 | -1.05 | -1.41 | -1.192 | **1.666** |
| SNORD114-16 | NR_003209 | 1.04 | -1.11 | -1.026 | **1.664** |
| TLN2 | NM_015059 | -1.04 | -1.32 | 1.007 | **1.664** |
| PNRC2 | NM_017761 | **2.85** | **1.96** | 1.426 | **1.663** |
| LOC105379520 | XR_951293 | 1.21 | **1.55** | **1.714** | **1.663** |
| MIR4295 | NR_036177 | 1.21 | **1.58** | -1.039 | **1.661** |
| MGC24103 | AK021795 | 1.21 | -1.01 | 1.226 | **1.661** |
| CTGF | NM_001901 | -1.12 | -1.27 | 1.294 | **1.661** |
| RAI14 | NM_001145520 | -1.06 | -1.30 | 1.259 | **1.661** |
| CARD8-AS1 | NR_040599 | 1.32 | 1.03 | **1.515** | **1.656** |
| LOC100268168 | NR_026682 | 1.01 | -1.18 | 1.212 | **1.655** |
| RGS2 | NM_002923 | -1.04 | -1.04 | **1.593** | **1.654** |
| LOC102724434 | NR_130921 | 1.30 | 1.45 | **2.387** | **1.654** |
| LOC101928389 | XR_247302 | 1.13 | -1.00 | -1.235 | **1.654** |
| LOC101928820 | XR_241868 | -1.50 | **-2.12** | -1.245 | **1.653** |
| CYP27C1 | NM_001001665 | 1.11 | -1.05 | 1.325 | **1.652** |
| LOC101927481 | NR_126336 | 1.21 | -1.22 | 1.114 | **1.646** |
| SNORD116-27 | NR_003341 | -1.38 | -1.13 | **2.385** | **1.644** |
| JARID2 | NM_001267040 | 1.12 | -1.12 | 1.315 | **1.644** |
| ZNF280D | OTTHUMT00000419442 | **1.65** | 1.29 | 1.087 | **1.643** |
| COL5A1 | NM_000093 | -1.17 | **-1.94** | -1.446 | **1.639** |
| ULBP3 | NM_024518 | 1.09 | -1.29 | **1.657** | **1.634** |
| LOC100507487 | NR_125882 | -1.16 | -1.26 | -1.227 | **1.633** |
| LOC101928161 | NR_110294 | 1.08 | 1.23 | **1.796** | **1.633** |
| MFAP3 | ENST00000519325 | -1.33 | -1.42 | 1.003 | **1.631** |
| PCDHB5 | NM_015669 | 1.41 | 1.29 | **2.043** | **1.629** |
| CEP170P1 | NR_003135 | -1.13 | -1.22 | 1.251 | **1.628** |
| MIR1269B | NR_039747 | **1.77** | **1.94** | **2.098** | **1.628** |
| SLC26A1 | NM_022042 | **1.53** | 1.23 | **1.692** | **1.625** |
| MIR3142 | NR_036095 | **1.69** | 1.07 | 1.363 | **1.625** |
| ITGB3 | NM_000212 | -1.20 | **-2.11** | **-1.643** | **1.624** |
| GAL | NM_015973 | 1.22 | -1.31 | 1.178 | **1.623** |
| MMP25-AS1 | NR_123723 | 1.41 | -1.02 | 1.021 | **1.623** |
| RHCG | NM_016321 | 1.33 | -1.15 | 1.074 | **1.622** |
| TENM3 | NM_001080477 | -1.01 | -1.09 | 1.003 | **1.622** |
| MIR1299 | NR_031629 | 1.35 | **1.94** | **1.568** | **1.622** |
| PYGL | NM_001163940 | -1.02 | -1.38 | 1.182 | **1.620** |
| STRA6 | NM_001142617 | 1.30 | -1.36 | -1.065 | **1.619** |
| SNORD114-2 | NR_003194 | 1.21 | -1.21 | 1.111 | **1.616** |
| GVQW1 | AK098413 | 1.32 | 1.25 | **1.688** | **1.613** |
| AQP7P3 | NR_026558 | **1.64** | 1.27 | 1.251 | **1.613** |
| SLC6A16 | NM_014037 | **1.53** | 1.22 | 1.419 | **1.612** |
| CTPS1 | NM_001301237 | -1.11 | **-1.52** | 1.070 | **1.611** |
| LOC105373343 | ENST00000458577 | **1.56** | 1.07 | 1.434 | **1.610** |
| ALPK2 | NM_052947 | **-1.55** | **-2.06** | -1.297 | **1.610** |
| CD59 | NM_000611 | 1.04 | -1.00 | 1.291 | **1.610** |
| PXN | NM_001080855 | 1.06 | -1.30 | 1.254 | **1.608** |
| MIR3122 | NR_036068 | **1.65** | 1.12 | 1.398 | **1.602** |
| CGB | NM_000737 | **1.57** | -1.15 | 1.148 | **1.602** |
| RBMS3 | NM_001003792 | -1.13 | -1.44 | -1.178 | **1.599** |
| C12orf56 | NM_001099676 | 1.18 | -1.04 | 1.305 | **1.599** |
| LOC101926984 | XR_918282 | **1.52** | 1.11 | 1.462 | **1.598** |
| LOC100506123 | uc021vll.1 | 1.35 | 1.17 | -1.010 | **1.598** |
| SNORD114-21 | NR_003214 | 1.03 | -1.19 | 1.304 | **1.597** |
| SNORD127 | NR_003691 | 1.36 | 1.10 | 1.429 | **1.595** |
| TBC1D22A-AS1 | NR_122047 | **1.76** | 1.23 | 1.288 | **1.594** |
| TAS2R10 | NM_023921 | 1.28 | -1.01 | -1.112 | **1.593** |
| SNORD114-14 | NR_003207 | 1.44 | 1.02 | 1.179 | **1.593** |
| MRC2 | NM_006039 | 1.12 | -1.17 | 1.382 | **1.593** |
| FOXP1 | NM_001012505 | -1.03 | -1.39 | -1.089 | **1.593** |
| MIR32 | NR_029506 | -1.06 | 1.19 | 1.188 | **1.592** |
| SLCO5A1 | NM_001146008 | 1.39 | 1.10 | 1.262 | **1.592** |
| LCE2C | NM_178429 | 1.48 | 1.23 | **1.578** | **1.591** |
| GPAM | NM_001244949 | 1.10 | -1.24 | 1.051 | **1.591** |
| LOC105369669 | XM_011508340 | **1.78** | 1.13 | 1.227 | **1.591** |
| OLFML3 | NM_001286352 | 1.36 | -1.34 | 1.378 | **1.589** |
| OR7E14P | NR_045002 | -1.38 | -1.43 | 1.044 | **1.588** |
| OCM2 | NM_006188 | 1.20 | 1.23 | **1.648** | **1.588** |
| METTL15 | XR_930849 | 1.13 | -1.04 | -1.012 | **1.586** |
| LOC105370924 | XR_916596 | -1.01 | -1.34 | -1.420 | **1.586** |
| MIR548L | NR_031630 | 1.16 | 1.08 | 1.053 | **1.586** |
| CEP162 | NM_001286206 | -1.25 | **-1.52** | 1.140 | **1.585** |
| MIR361 | NR_029848 | **1.59** | 1.42 | **1.551** | **1.585** |
| MT1F | NM_001301272 | 1.05 | -1.17 | 1.237 | **1.585** |
| BVES | NM_001199563 | -1.23 | **-1.98** | -1.239 | **1.584** |
| SAPCD1 | NM_001039651 | 1.39 | 1.12 | 1.279 | **1.583** |
| HEPN1 | NM_001037558 | **1.69** | 1.19 | 1.282 | **1.583** |
| C10orf113 | NM_001010896 | 1.05 | -1.03 | 1.239 | **1.582** |
| BLM | NM_000057 | -1.22 | **-1.91** | -1.005 | **1.582** |
| SLC22A4 | NM_003059 | 1.14 | **-1.62** | -1.225 | **1.578** |
| MIR544A | NR_030257 | 1.23 | 1.07 | **1.618** | **1.578** |
| MIR1207 | NR_031612 | 1.18 | 1.31 | 1.072 | **1.577** |
| LOC645513 | NR_037630 | 1.17 | -1.07 | **1.533** | **1.577** |
| C3orf67 | NM_198463 | 1.09 | -1.27 | -1.019 | **1.576** |
| MIR4677 | NR_039824 | 1.21 | 1.31 | 1.168 | **1.576** |
| RHOD | NM_001300886 | 1.14 | -1.14 | 1.225 | **1.575** |
| MTMR11 | NM_001145862 | 1.06 | -1.32 | 1.005 | **1.574** |
| ZNF43 | NM_001256648 | 1.30 | -1.19 | 1.106 | **1.574** |
| LOC105369370 | XR_913267 | 1.14 | -1.10 | 1.289 | **1.574** |
| SLC6A15 | NM_001146335 | **1.75** | 1.09 | **1.786** | **1.574** |
| LTBP2 | NM_000428 | 1.13 | -1.20 | 1.139 | **1.573** |
| CNGA2 | NM_005140 | **1.80** | 1.05 | 1.097 | **1.573** |
| ANXA8 | AK296976 | **-1.80** | **-3.19** | **-1.687** | **1.570** |
| SPDL1 | NM_017785 | -1.48 | **-2.26** | -1.172 | **1.570** |
| MIR501 | NR_030225 | **1.97** | **2.01** | **1.874** | **1.569** |
| MIR365B | NR_029856 | **1.75** | 1.11 | 1.070 | **1.569** |
| E2F3 | XM_011514327 | -1.17 | -1.09 | 1.198 | **1.568** |
| MOB3B | NM_024761 | 1.01 | -1.49 | 1.080 | **1.568** |
| MYO10 | NM_012334 | 1.04 | -1.17 | 1.131 | **1.568** |
| ARHGAP31 | NM_020754 | 1.14 | -1.17 | 1.144 | **1.567** |
| PIGL | NM_004278 | 1.13 | -1.12 | 1.240 | **1.567** |
| MS4A6A | NM_001247999 | **1.50** | 1.06 | **1.709** | **1.566** |
| RUSC2 | NM_001135999 | -1.22 | -1.31 | 1.128 | **1.566** |
| TREM1 | NM_001242589 | 1.30 | 1.09 | 1.348 | **1.565** |
| CD83 | NM_001040280 | 1.28 | 1.04 | **1.637** | **1.565** |
| LOC101928152 | XR_245037 | 1.06 | -1.01 | **1.536** | **1.563** |
| IGHA1 | AF067420 | 1.30 | -1.10 | 1.190 | **1.563** |
| MIR550A3 | NR_039600 | **1.57** | 1.23 | 1.245 | **1.563** |
| NUAK2 | NM_030952 | -1.08 | **-1.51** | 1.213 | **1.562** |
| PACSIN3 | NM_001184974 | 1.13 | -1.30 | 1.166 | **1.562** |
| RAET1G | NM_001001788 | -1.04 | -1.12 | 1.048 | **1.561** |
| ZNF827 | NM_001306215 | 1.09 | -1.24 | 1.121 | **1.560** |
| TAS2R30 | ENST00000539585 | 1.34 | **1.61** | 1.081 | **1.560** |
| CLTCL1 | NM_001835 | -1.05 | -1.46 | 1.120 | **1.557** |
| ANXA8 | NM_001040084 | **-1.81** | **-3.11** | **-1.659** | **1.557** |
| YTHDC1 | ENST00000506175 | -1.16 | 1.09 | 1.251 | **1.554** |
| LOC105375236 | XR_927180 | 1.02 | -1.10 | 1.086 | **1.554** |
| MIR513A2 | NR_030232 | 1.07 | 1.45 | **1.850** | **1.553** |
| LINC01032 | NR_126003 | 1.18 | -1.18 | -1.033 | **1.552** |
| TRAJ16 | OTTHUMT00000410982 | **3.25** | 1.41 | 1.337 | **1.549** |
| SNORD114-31 | NR_003224 | -1.06 | **-1.53** | -1.042 | **1.549** |
| LOC105375451 | XR_927863 | 1.24 | 1.08 | 1.277 | **1.547** |
| SPACA5 | uc004diu.3 | **1.54** | 1.12 | 1.404 | **1.545** |
| SNORA46 | NR_002978 | 1.39 | **2.57** | **2.362** | **1.544** |
| HSPB1 | NM_001540 | -1.30 | **-1.69** | -1.122 | **1.544** |
| LOC105372966 | XR_920883 | -1.07 | -1.49 | 1.082 | **1.543** |
| OLFM2 | NM_001304347 | 1.11 | -1.05 | 1.365 | **1.543** |
| LOC102724196 | XR_429051 | 1.11 | -1.03 | 1.154 | **1.543** |
| MIR4529 | NR_039754 | 1.45 | 1.43 | **1.678** | **1.543** |
| LINC01355 | NR_110616 | -1.02 | **-1.52** | -1.244 | **1.542** |
| MIR1305 | NR_031640 | -1.28 | -1.37 | 1.000 | **1.542** |
| SHANK2 | ENST00000608988 | 1.19 | 1.34 | **1.583** | **1.541** |
| MIR4768 | NR_039925 | -1.05 | -1.43 | -1.291 | **1.541** |
| KIRREL3 | NM_001161707 | -1.24 | **-1.78** | 1.043 | **1.539** |
| NDUFAF6 | NM_152416 | 1.14 | 1.05 | 1.467 | **1.539** |
| FAT3 | NM_001008781 | 1.14 | -1.30 | -1.113 | **1.538** |
| PHLDA1 | NM_007350 | 1.38 | 1.08 | 1.485 | **1.538** |
| MT1CP | OTTHUMT00000434321 | 1.13 | **-1.57** | -1.362 | **1.537** |
| MIR4684 | NR_039832 | 1.19 | 1.02 | 1.132 | **1.535** |
| CNKSR2 | NM_001168647 | 1.11 | -1.30 | 1.230 | **1.535** |
| ST8SIA1 | NM_001304450 | 1.42 | 1.01 | 1.228 | **1.534** |
| VOPP1 | NM_001284282 | 1.12 | -1.05 | 1.152 | **1.534** |
| EDIL3 | NM_001278642 | 1.21 | 1.03 | 1.209 | **1.534** |
| ELK3 | NM_001303511 | -1.06 | -1.33 | 1.118 | **1.534** |
| C7orf73 | NM_001130929 | 1.01 | -1.19 | -1.061 | **1.533** |
| PLA2R1 | NM_001007267 | 1.07 | -1.16 | -1.015 | **1.533** |
| MIR4324 | NR_036209 | 1.40 | 1.19 | **1.527** | **1.532** |
| LOC100506458 | AK097034 | 1.06 | -1.15 | -1.165 | **1.532** |
| TAS2R50 | NM_176890 | 1.19 | 1.18 | 1.032 | **1.532** |
| SLAMF9 | NM_001146172 | 1.26 | 1.05 | 1.185 | **1.531** |
| USP17L10 | NM_001256852 | 1.32 | 1.17 | **1.651** | **1.529** |
| GPX3 | NM_002084 | 1.23 | -1.07 | **1.711** | **1.529** |
| PRDM1 | NM_001198 | **1.54** | -1.06 | 1.167 | **1.528** |
| TAS2R14 | NM_023922 | -1.17 | 1.46 | 1.172 | **1.527** |
| NLRP1 | ENST00000544378 | 1.19 | 1.08 | 1.183 | **1.526** |
| LOC105375032 | XR_926742 | 1.09 | 1.11 | 1.367 | **1.526** |
| PRICKLE2 | NM_198859 | 1.35 | 1.12 | 1.408 | **1.525** |
| IGBP1P1 | NR_002937 | **2.26** | **1.79** | **1.853** | **1.525** |
| SEPT6 | NM_015129 | 1.40 | 1.11 | **1.746** | **1.524** |
| ZNF462 | NM_021224 | 1.42 | -1.10 | **1.566** | **1.524** |
| SNORD114-30 | NR_003223 | 1.00 | -1.47 | -1.499 | **1.522** |
| SNORA65 | NR_002449 | 1.24 | **1.77** | -1.005 | **1.522** |
| GAREM2 | NM_001168241 | 1.28 | -1.06 | 1.214 | **1.522** |
| MIR4796 | NR_039959 | 1.17 | 1.36 | 1.436 | **1.522** |
| LINC00312 | NR_024065 | **-1.79** | **-2.02** | -1.070 | **1.521** |
| DCBLD1 | NM_173674 | -1.36 | -1.40 | 1.026 | **1.521** |
| GADD45B | NM_015675 | -1.04 | 1.11 | 1.120 | **1.521** |
| ZNF185 | NM_001178106 | **-1.57** | **-2.00** | -1.376 | **1.519** |
| PRAMEF11 | NM_001146344 | **2.02** | **1.52** | **1.644** | **1.518** |
| TSPAN16 | NM_001282509 | 1.13 | 1.00 | 1.306 | **1.518** |
| PTPRB | NM_001109754 | 1.21 | 1.14 | **1.542** | **1.517** |
| LPAR5 | NM_001142961 | 1.09 | -1.14 | 1.043 | **1.516** |
| MIR320E | NR_036157 | **1.51** | 1.17 | 1.413 | **1.515** |
| TSHZ3 | NM_020856 | -1.35 | -1.41 | -1.055 | **1.515** |
| PORCN | NM_001282167 | 1.24 | -1.05 | 1.296 | **1.514** |
| CREG2 | NM_153836 | 1.32 | 1.07 | 1.192 | **1.514** |
| PDGFB | NM_002608 | -1.02 | -1.27 | 1.147 | **1.514** |
| PPP1R14C | NM_030949 | -1.29 | **-1.58** | -1.104 | **1.514** |
| NHS | NM_001136024 | -1.19 | -1.15 | 1.215 | **1.513** |
| ADA | NM_000022 | 1.12 | -1.15 | 1.447 | **1.513** |
| C8orf17 | AF220264 | **1.76** | 1.11 | 1.240 | **1.512** |
| SNORA70B | NR_003707 | -1.00 | 1.18 | 1.443 | **1.512** |
| HAS2 | NM_005328 | 1.15 | -1.19 | 1.027 | **1.512** |
| LPAR3 | NM_012152 | **1.51** | 1.12 | 1.253 | **1.512** |
| TMEM132D | NM_133448 | 1.49 | 1.10 | **1.602** | **1.512** |
| CDRT15P2 | NR_033865 | 1.19 | 1.20 | 1.340 | **1.510** |
| MIR101-1 | NR_029516 | 1.29 | 1.35 | **1.540** | **1.510** |
| GFRA1 | NM_001145453 | **1.57** | -1.19 | 1.301 | **1.510** |
| ANKRD36C | NM_001310154 | 1.06 | -1.23 | -1.082 | **1.509** |
| SDHA | ENST00000502379 | **1.58** | **1.75** | 1.328 | **1.509** |
| BLOC1S2 | NM_001001342 | -1.27 | -1.50 | 1.009 | **1.507** |
| LOC101929506 | XR_246145 | -1.03 | -1.00 | 1.057 | **1.507** |
| MIR563 | NR_030289 | **1.62** | 1.36 | 1.403 | **1.506** |
| DBN1 | NM_004395 | 1.15 | -1.14 | 1.257 | **1.506** |
| TRAV1-2 | OTTHUMT00000401873 | 1.29 | -1.02 | 1.251 | **1.506** |
| FLJ21369 | AK025022 | 1.11 | 1.09 | 1.380 | **1.505** |
| GLIPR1 | NM_006851 | **-1.74** | **-2.57** | **-1.859** | **1.504** |
| LOC100287934 | XR_108279 | 1.45 | 1.29 | **1.543** | **1.504** |
| ANXA8L1 | OTTHUMT00000047846 | 1.18 | -1.01 | 1.265 | **1.503** |
| FAM101B | NM_182705 | 1.38 | 1.15 | **1.807** | **1.503** |
| SMAD4 | ENST00000585448 | 1.10 | 1.11 | 1.497 | **1.501** |
| UCP2 | NM_003355 | 1.13 | -1.22 | 1.299 | **1.501** |
| E2F7 | NM_203394 | -1.32 | **-2.05** | -1.351 | **1.501** |
| LOC286437 | NR_039980 | 1.36 | -1.02 | 1.046 | **1.500** |
| LOC100130502 | XR_244997 | **-1.58** | **-1.55** | 1.067 | **1.500** |
| ULBP2 | NM_025217 | 1.00 | -1.05 | **1.948** | **1.500** |
| BTN3A1 | NM_001145008 | **-2.16** | -1.48 | -1.287 | **-1.500** |
| CABLES1 | NM_001100619 | -1.09 | 1.31 | 1.075 | **-1.501** |
| LOC100101148 | NR_104007 | -1.43 | 1.11 | 1.163 | **-1.503** |
| GSTA2 | NM_000846 | **-1.60** | -1.10 | -1.228 | **-1.503** |
| HGD | NM_000187 | -1.41 | 1.00 | **-1.774** | **-1.504** |
| CCDC68 | NM_001143829 | -1.49 | 1.28 | -1.140 | **-1.505** |
| LOC100652999 | NR_046221 | -1.48 | -1.25 | -1.476 | **-1.505** |
| LOC105369871 | XR_914305 | **-1.59** | -1.26 | **-1.835** | **-1.506** |
| MIR657 | NR_030394 | -1.48 | -1.43 | **-1.775** | **-1.506** |
| C4orf32 | NM_152400 | -1.18 | 1.09 | -1.195 | **-1.507** |
| IRF2BP2 | NM_001077397 | -1.03 | 1.25 | -1.013 | **-1.507** |
| HNF4G | NM_004133 | -1.30 | -1.10 | -1.456 | **-1.508** |
| AREG | ENST00000511560 | 1.14 | 1.18 | 1.043 | **-1.509** |
| SMR3A | NM_012390 | **-1.60** | -1.03 | 1.066 | **-1.509** |
| NCR3LG1 | NM_001202439 | -1.24 | 1.14 | -1.448 | **-1.510** |
| TRAJ58 | OTTHUMT00000410940 | **-2.70** | -1.26 | **-1.588** | **-1.510** |
| MIR548V | NR_036103 | 1.11 | **2.04** | -1.309 | **-1.510** |
| EMP1 | NM_001423 | -1.40 | 1.21 | 1.024 | **-1.511** |
| EREG | NM_001432 | **1.59** | 1.33 | 1.447 | **-1.513** |
| CCAT1 | NR_108049 | -1.41 | 1.23 | -1.479 | **-1.513** |
| IGHV3-38 | OTTHUMT00000325190 | -1.23 | 1.05 | -1.444 | **-1.514** |
| TMTC2 | NM_152588 | -1.20 | 1.20 | -1.040 | **-1.514** |
| LOC105376694 | XR_931876 | -1.01 | -1.15 | **-1.859** | **-1.515** |
| MTUS1 | NM_001001924 | -1.12 | **1.85** | -1.076 | **-1.516** |
| LOC105372352 | XR_919562 | -1.22 | -1.25 | **-2.045** | **-1.516** |
| KLRC3 | NM_002261 | -1.13 | **1.52** | 1.230 | **-1.517** |
| TAT | NM_000353 | -1.38 | -1.09 | -1.336 | **-1.517** |
| KLHL4 | NM_019117 | **-1.56** | -1.33 | -1.207 | **-1.518** |
| ELF3 | NM_001114309 | -1.20 | 1.01 | -1.306 | **-1.518** |
| LOC105372845 | XR_920599 | -1.14 | 1.40 | -1.120 | **-1.518** |
| RASEF | NM_152573 | **-1.56** | 1.03 | -1.498 | **-1.520** |
| TIGD2 | NM_145715 | **-1.71** | -1.07 | -1.254 | **-1.521** |
| KLF9 | NM_001206 | -1.03 | 1.14 | -1.058 | **-1.521** |
| SLITRK6 | NM_032229 | -1.10 | 1.15 | -1.013 | **-1.523** |
| RHOBTB3 | NM_014899 | 1.02 | 1.39 | -1.021 | **-1.523** |
| LOC101927629 | XR_243458 | -1.45 | -1.06 | -1.438 | **-1.523** |
| APOL6 | NM_030641 | -1.50 | -1.47 | **-1.802** | **-1.523** |
| PLA1A | NM_001206960 | -1.39 | -1.04 | **-1.571** | **-1.527** |
| KIAA1147 | NM_001080392 | **-1.62** | 1.06 | **-1.536** | **-1.527** |
| GAREM1 | NM_001242409 | -1.48 | 1.02 | -1.249 | **-1.527** |
| JADE2 | NM_001289984 | -1.29 | 1.06 | -1.131 | **-1.528** |
| ADH4 | NM_000670 | -1.17 | -1.03 | -1.392 | **-1.528** |
| DNAPTP3 | AK094277 | **-1.68** | **-1.59** | **-1.730** | **-1.528** |
| MGC2889 | NR_026877 | -1.12 | -1.12 | **-1.815** | **-1.528** |
| MAPRE2 | NM_001143826 | -1.06 | 1.24 | -1.008 | **-1.528** |
| LOC101929518 | XR_252569 | -1.47 | 1.20 | -1.276 | **-1.529** |
| LOC105372331 | XR_919526 | -1.48 | -1.39 | **-1.557** | **-1.529** |
| THEM5 | NM_182578 | -1.17 | -1.06 | **-1.572** | **-1.530** |
| TRAV1-1 | OTTHUMT00000401872 | -1.26 | 1.01 | -1.326 | **-1.530** |
| SLC7A2 | NM_001008539 | **1.68** | **2.91** | 1.451 | **-1.531** |
| TRBV5-3 | OTTHUMT00000485125 | **-1.56** | -1.18 | -1.366 | **-1.532** |
| STS | NM_000351 | **-1.57** | -1.19 | **-1.810** | **-1.532** |
| HEXDC-IT1 | AK129961 | **-1.56** | -1.50 | **-2.030** | **-1.532** |
| LRRC8D | NM_001134479 | **-1.56** | 1.03 | -1.102 | **-1.533** |
| CDKN2C | NM_001262 | **-1.79** | -1.09 | **-1.674** | **-1.533** |
| ZBED3 | NM_032367 | -1.01 | 1.03 | -1.394 | **-1.533** |
| THAP3 | NM_001195752 | **-1.53** | -1.13 | -1.250 | **-1.535** |
| FCGBP | NM_003890 | -1.14 | 1.05 | **-1.614** | **-1.535** |
| IFI30 | NM_006332 | -1.10 | 1.23 | -1.112 | **-1.537** |
| TBC1D30 | NM_015279 | -1.13 | 1.04 | -1.339 | **-1.538** |
| ATP8B1 | NM_005603 | **-1.61** | -1.17 | -1.357 | **-1.538** |
| SLC6A14 | NM_007231 | 1.23 | **1.71** | 1.328 | **-1.538** |
| CXCL16 | NM_001100812 | -1.18 | 1.18 | 1.067 | **-1.539** |
| LINC01065 | NR_125788 | **-1.55** | -1.13 | -1.387 | **-1.539** |
| EPB41L4A | NM_022140 | **-2.01** | -1.27 | **-1.726** | **-1.539** |
| AIM1 | NM_001624 | **-1.68** | -1.14 | -1.124 | **-1.540** |
| PAPSS2 | NM_001015880 | -1.12 | 1.42 | -1.098 | **-1.540** |
| IGHV3-72 | OTTHUMT00000324210 | -1.13 | -1.03 | **-1.625** | **-1.540** |
| TPD52L3 | NM_001001874 | -1.24 | -1.11 | **-1.614** | **-1.541** |
| BCL6 | NM_001130845 | 1.12 | **1.64** | -1.063 | **-1.542** |
| TRPC6 | NM_004621 | -1.34 | 1.03 | -1.056 | **-1.542** |
| KCNT2 | NM_001287819 | -1.31 | -1.26 | **-1.933** | **-1.543** |
| TFPI | NM_001032281 | -1.48 | 1.07 | -1.267 | **-1.546** |
| MIR601 | NR_030332 | -1.46 | -1.03 | -1.009 | **-1.548** |
| IRX5 | NM_001252197 | **-1.62** | -1.28 | -1.500 | **-1.548** |
| JMY | NM_152405 | -1.05 | 1.43 | 1.018 | **-1.549** |
| CSF2RA | NM_001161529 | -1.40 | -1.33 | -1.221 | **-1.549** |
| PLCD4 | NM_032726 | **-1.65** | -1.06 | -1.285 | **-1.550** |
| RGS21 | NM_001039152 | -1.33 | -1.07 | **-1.599** | **-1.551** |
| IFNGR1 | NM_000416 | -1.05 | 1.43 | 1.065 | **-1.551** |
| LOC105375112 | XM_011515664 | -1.28 | -1.06 | -1.464 | **-1.551** |
| GBE1 | NM_000158 | -1.33 | 1.04 | -1.285 | **-1.551** |
| IGFBP1 | NM_000596 | 1.30 | 1.22 | -1.152 | **-1.552** |
| FCGR3A | NM_000569 | **-1.87** | -1.41 | **-2.328** | **-1.552** |
| CYP4Z1 | NM_178134 | -1.22 | -1.03 | **-1.683** | **-1.552** |
| PDE3A | NM_000921 | **-1.84** | 1.25 | **-1.826** | **-1.553** |
| SHROOM3 | XM_011532157 | **-1.57** | -1.34 | -1.428 | **-1.555** |
| CASP4 | NM_001225 | -1.40 | -1.01 | -1.255 | **-1.557** |
| DBP | NM_001352 | 1.03 | 1.20 | -1.086 | **-1.557** |
| MIR548Y | NR_037503 | -1.29 | 1.07 | -1.409 | **-1.559** |
| ANGPT1 | NM_001146 | -1.41 | 1.01 | -1.276 | **-1.559** |
| CYP2A13 | NM_000766 | -1.38 | 1.17 | -1.171 | **-1.561** |
| SNORD14E | NR_003125 | **-1.66** | 1.19 | -1.001 | **-1.562** |
| STAT4 | NM_001243835 | -1.02 | 1.05 | 1.146 | **-1.566** |
| OSGIN1 | NM_182981 | 1.08 | 1.15 | -1.248 | **-1.568** |
| MUC5B | NM_002458 | 1.16 | **2.04** | -1.013 | **-1.570** |
| CLMN | NM_024734 | -1.40 | -1.00 | -1.314 | **-1.572** |
| SLC51B | NM_178859 | -1.01 | 1.27 | -1.211 | **-1.572** |
| FKBP5 | NM_001145775 | -1.25 | -1.06 | -1.260 | **-1.574** |
| SNTB1 | NM_021021 | -1.20 | -1.00 | -1.333 | **-1.575** |
| EPS8 | NM_004447 | -1.46 | -1.01 | -1.418 | **-1.577** |
| ALX1 | NM_006982 | **-1.77** | -1.07 | -1.164 | **-1.578** |
| SLC23A2 | NM_005116 | 1.03 | **1.66** | -1.075 | **-1.578** |
| MIR4287 | NR_036249 | **-1.70** | -1.23 | **-1.712** | **-1.578** |
| FAM214A | NM_001286495 | -1.25 | 1.18 | -1.304 | **-1.580** |
| DNAJC12 | NM_021800 | 1.00 | **1.65** | -1.059 | **-1.581** |
| RAB38 | NM_022337 | -1.25 | -1.05 | -1.208 | **-1.586** |
| PGM5 | NM_021965 | -1.19 | -1.07 | **-1.598** | **-1.586** |
| LRRC37A6P | NR_003525 | -1.29 | -1.02 | -1.349 | **-1.589** |
| IGKV1D-27 | OTTHUMT00000323279 | 1.08 | 1.13 | -1.477 | **-1.591** |
| RAB40B | NM_006822 | -1.33 | 1.04 | -1.414 | **-1.592** |
| HIST1H2AJ | NM_021066 | **-1.64** | **-1.81** | **-1.968** | **-1.592** |
| IL1R2 | NM_001261419 | -1.42 | 1.08 | -1.353 | **-1.592** |
| TOX3 | NM_001080430 | **-1.54** | -1.25 | **-1.751** | **-1.595** |
| LOC283710 | NM_001243538 | **-1.56** | 1.05 | -1.379 | **-1.596** |
| HNRNPA1L2 | NM_001011724 | **-2.46** | **-1.67** | -1.316 | **-1.599** |
| MIR4678 | NR_039825 | 1.18 | 1.24 | -1.002 | **-1.599** |
| MIR4782 | NR_039943 | **-1.71** | -1.24 | -1.171 | **-1.600** |
| MIR200B | NR_029639 | **-1.55** | -1.30 | **-1.720** | **-1.600** |
| PMP22 | NM_000304 | -1.01 | 1.44 | 1.040 | **-1.601** |
| ARRB2 | NM_001257328 | -1.05 | 1.24 | -1.065 | **-1.602** |
| TDP2 | NM_016614 | -1.19 | -1.04 | **-1.554** | **-1.602** |
| CES1 | NM_001025194 | -1.18 | 1.07 | -1.310 | **-1.602** |
| LOC105378749 | XR_947404 | -1.35 | -1.04 | **-1.501** | **-1.603** |
| PGM5P4-AS1 | NR_121185 | -1.34 | 1.07 | **-1.525** | **-1.603** |
| WHAMM | NM_001080435 | **-1.89** | -1.30 | -1.479 | **-1.604** |
| GATS | NM_178831 | -1.49 | 1.11 | -1.225 | **-1.609** |
| SLC12A2 | NM_001046 | 1.23 | **1.84** | -1.059 | **-1.609** |
| FSTL4 | NM_015082 | 1.48 | **2.11** | 1.004 | **-1.613** |
| LUNAR1 | NR_126487 | -1.15 | -1.06 | -1.295 | **-1.613** |
| CYP4F11 | NM_001128932 | -1.29 | -1.21 | -1.426 | **-1.614** |
| CPT1A | NM_001031847 | -1.40 | 1.13 | -1.225 | **-1.614** |
| THSD7A | NM_015204 | -1.27 | 1.14 | -1.278 | **-1.616** |
| POF1B | NM_001307940 | -1.50 | -1.11 | **-1.565** | **-1.618** |
| NOV | NM_002514 | -1.09 | 1.28 | -1.148 | **-1.624** |
| OR52M1 | NM_001004137 | **-1.54** | -1.31 | **-1.687** | **-1.627** |
| SVEP1 | NM_153366 | -1.33 | **1.50** | -1.100 | **-1.628** |
| TRAJ44 | OTTHUMT00000410954 | -1.40 | -1.29 | **-1.691** | **-1.631** |
| ALDH3A1 | NM_000691 | 1.02 | 1.20 | 1.099 | **-1.632** |
| CEACAM1 | NM_001024912 | -1.19 | 1.20 | **-1.714** | **-1.635** |
| ARHGAP18 | NM_033515 | **-1.54** | 1.11 | -1.220 | **-1.641** |
| BTN3A3 | NM_001242803 | 1.00 | 1.21 | -1.313 | **-1.642** |
| LINC00240 | NR_026775 | -1.48 | 1.01 | -1.067 | **-1.642** |
| GLYCAM1 | NR_003039 | **-1.69** | -1.13 | -1.461 | **-1.644** |
| CRLF2 | uc022brs.1 | -1.29 | 1.07 | -1.190 | **-1.652** |
| TMEM133 | NM_032021 | **-2.05** | -1.06 | -1.302 | **-1.653** |
| CACNA1D | NM_000720 | 1.06 | **1.75** | -1.178 | **-1.661** |
| GPX2 | NM_002083 | -1.33 | 1.08 | **-1.513** | **-1.661** |
| LINC00680 | NR_125727 | -1.34 | 1.43 | **-1.618** | **-1.664** |
| TNFRSF9 | NM_001561 | -1.36 | **-1.54** | -1.441 | **-1.668** |
| MIR548AB | NR_039611 | -1.46 | -1.07 | -1.288 | **-1.669** |
| IGHV3-42 | OTTHUMT00000325176 | -1.38 | -1.04 | -1.487 | **-1.669** |
| RGL1 | NM_001297669 | **-1.86** | -1.21 | **-1.654** | **-1.675** |
| MIR616 | NR_030346 | -1.40 | -1.17 | -1.273 | **-1.676** |
| DOCK11 | NM_144658 | -1.26 | 1.07 | -1.331 | **-1.680** |
| GALNT5 | NM_014568 | **-1.65** | 1.05 | -1.251 | **-1.682** |
| PLEKHH2 | NM_172069 | 1.23 | **1.67** | -1.255 | **-1.685** |
| ABCG2 | NM_001257386 | -1.41 | 1.01 | -1.224 | **-1.687** |
| ZNF888 | NM_001310127 | **-1.99** | -1.18 | **-1.673** | **-1.689** |
| ANKS4B | NM_145865 | -1.45 | -1.04 | **-2.125** | **-1.690** |
| FOS | NM_005252 | -1.01 | **3.14** | 1.160 | **-1.691** |
| CYP2S1 | NM_030622 | 1.03 | 1.29 | -1.212 | **-1.692** |
| PKDCC | NM_138370 | -1.48 | -1.10 | **-1.550** | **-1.693** |
| TMC5 | NM_001105248 | **-1.51** | 1.16 | -1.143 | **-1.693** |
| PLA2G4A | NM_001311193 | 1.10 | **1.93** | 1.001 | **-1.696** |
| CDC25B | NM_001287516 | 1.03 | 1.29 | -1.103 | **-1.698** |
| CES1P1 | ENST00000421606 | -1.21 | 1.01 | -1.367 | **-1.699** |
| CYP3A5 | NM_000777 | -1.46 | -1.18 | **-1.561** | **-1.700** |
| CYP1B1 | NM_000104 | 1.08 | 1.07 | 1.040 | **-1.703** |
| PLCXD3 | NM_001005473 | **-1.74** | -1.09 | **-1.587** | **-1.704** |
| ACSM3 | NM_005622 | -1.08 | 1.09 | -1.042 | **-1.708** |
| VTCN1 | NM_001253849 | -1.33 | 1.00 | **-1.752** | **-1.710** |
| LOC105370503 | XR_915628 | **-1.88** | -1.47 | **-1.870** | **-1.713** |
| CFB | NM_001710 | -1.46 | -1.15 | **-1.898** | **-1.717** |
| RAP1GAP | NM_001145657 | -1.30 | 1.17 | -1.119 | **-1.718** |
| PIK3C2B | NM_002646 | -1.31 | 1.30 | -1.257 | **-1.718** |
| ERN1 | NM_001433 | -1.08 | 1.15 | -1.413 | **-1.720** |
| RNF5P1 | NR_003129 | **-1.93** | -1.43 | **-1.595** | **-1.731** |
| HLF | NM_002126 | 1.07 | 1.30 | -1.272 | **-1.735** |
| GOLGA6L4 | NM_001267536 | -1.05 | -1.13 | **-1.869** | **-1.738** |
| PRR15 | NM_025014 | **-1.56** | 1.03 | -1.406 | **-1.747** |
| IL6R | NM_000565 | -1.38 | 1.15 | -1.045 | **-1.748** |
| MUC5AC | uc001lsz.3 | 1.12 | **1.64** | 1.067 | **-1.749** |
| PARM1 | NM_015393 | -1.26 | 1.38 | -1.156 | **-1.749** |
| VTRNA1-1 | NR_026703 | **-3.05** | -1.24 | **-1.825** | **-1.750** |
| AHR | NM_001621 | -1.17 | 1.27 | -1.235 | **-1.764** |
| ENPP1 | NM_006208 | -1.04 | 1.31 | 1.046 | **-1.766** |
| IFITM3 | NM_021034 | **-1.58** | 1.16 | -1.451 | **-1.769** |
| VTRNA1-3 | NR_026705 | -1.27 | -1.29 | **-1.863** | **-1.770** |
| CA12 | NM_001218 | -1.12 | 1.25 | -1.170 | **-1.774** |
| GATSL2 | NM_001145064 | **-1.60** | 1.07 | -1.215 | **-1.788** |
| PMCHL1 | NR_003921 | -1.32 | 1.46 | -1.053 | **-1.801** |
| SH3BGRL2 | NR_038227 | **-1.66** | 1.05 | -1.448 | **-1.802** |
| F5 | NM_000130 | -1.30 | 1.15 | **-1.718** | **-1.806** |
| SPDEF | XR_241133 | -1.36 | 1.13 | **-1.636** | **-1.806** |
| MMP7 | NM_002423 | **-1.53** | **-1.53** | **-2.086** | **-1.816** |
| IQGAP2 | NM_001285460 | -1.27 | **1.52** | -1.072 | **-1.821** |
| ADH6 | NM_000672 | **-1.53** | -1.27 | **-2.168** | **-1.821** |
| GDF15 | NM_004864 | **1.77** | **1.97** | 1.389 | **-1.824** |
| GLP2R | NM_004246 | **-1.53** | -1.00 | **-1.517** | **-1.829** |
| FER1L6 | NM_001039112 | **-1.80** | -1.40 | **-1.940** | **-1.832** |
| NR1H4 | NM_001206977 | **-1.89** | -1.42 | **-2.199** | **-1.841** |
| SLC27A2 | NM_001159629 | -1.43 | 1.11 | -1.499 | **-1.843** |
| C5 | NM_001735 | -1.01 | 1.36 | -1.323 | **-1.848** |
| ANG | NM_001097577 | -1.07 | 1.49 | -1.280 | **-1.859** |
| NR4A2 | NM_006186 | 1.01 | **3.06** | 1.132 | **-1.862** |
| CNTN1 | NM_001256063 | -1.03 | **1.50** | -1.138 | **-1.862** |
| RAB27B | NM_004163 | -1.37 | 1.10 | -1.411 | **-1.864** |
| FAM66B | NR_027423 | -1.25 | 1.13 | **-2.377** | **-1.869** |
| SGPP2 | NM_152386 | -1.48 | 1.01 | -1.242 | **-1.877** |
| IRS2 | NM_003749 | 1.02 | **2.11** | 1.184 | **-1.891** |
| GULP1 | NM_001252668 | -1.03 | **1.62** | -1.044 | **-1.908** |
| NR0B1 | NM_000475 | -1.01 | 1.23 | -1.097 | **-1.933** |
| GPCPD1 | NM_019593 | 1.18 | **2.09** | 1.163 | **-1.935** |
| TSPAN7 | NM_004615 | -1.46 | -1.28 | **-1.671** | **-1.950** |
| ITIH2 | NM_002216 | **-1.84** | -1.40 | **-2.704** | **-1.956** |
| PDE8B | NM_001029851 | **-1.50** | 1.26 | -1.351 | **-1.972** |
| KIR2DL2 | NM_014219 | -1.44 | -1.15 | **-2.156** | **-1.980** |
| SLC16A6 | NM_001174166 | **1.65** | **3.81** | **1.976** | **-1.984** |
| CFH | NM_000186 | -1.44 | 1.12 | **-1.861** | **-1.985** |
| TM4SF4 | NM_004617 | **-1.86** | -1.07 | **-2.103** | **-1.989** |
| SLPI | NM_003064 | -1.13 | 1.29 | -1.410 | **-1.992** |
| INHBB | NM_002193 | -1.33 | -1.00 | -1.161 | **-2.025** |
| SERPINB4 | NM_002974 | **-2.64** | **-1.54** | **-1.882** | **-2.025** |
| A1CF | NM_001198818 | **-1.79** | -1.29 | **-1.892** | **-2.035** |
| SLC40A1 | NM_014585 | **-1.95** | -1.03 | **-2.050** | **-2.048** |
| PDK4 | NM_002612 | **2.38** | **4.49** | **1.945** | **-2.053** |
| VTRNA1-2 | NR_026704 | **-3.82** | 1.01 | -1.430 | **-2.067** |
| CATSPERB | NM_024764 | -1.25 | **2.24** | **-1.709** | **-2.082** |
| KITLG | NM_000899 | -1.22 | 1.45 | -1.226 | **-2.094** |
| CYP4F3 | NM_000896 | **-1.59** | -1.07 | **-1.601** | **-2.108** |
| RHOBTB1 | NM_001242359 | -1.22 | 1.50 | **-1.605** | **-2.117** |
| ST8SIA4 | NM_005668 | -1.25 | 1.24 | -1.386 | **-2.131** |
| HABP2 | NM_001177660 | **-1.71** | -1.13 | **-2.474** | **-2.158** |
| PER2 | NM_022817 | -1.28 | 1.33 | -1.115 | **-2.163** |
| CDH1 | NM_004360 | **-1.52** | -1.02 | **-1.544** | **-2.172** |
| OR51E1 | NM_152430 | **-2.15** | -1.18 | **-3.192** | **-2.179** |
| MIR548I2 | NR_031688 | **-1.51** | -1.07 | **-1.997** | **-2.195** |
| MIA2 | NM_054024 | **-1.73** | -1.18 | **-2.308** | **-2.208** |
| SPX | NM_030572 | 1.12 | 1.30 | -1.290 | **-2.218** |
| FA2H | NM_024306 | -1.36 | 1.13 | -1.237 | **-2.235** |
| COLCA2 | NM_001136105 | **-1.62** | 1.03 | **-2.470** | **-2.248** |
| TC2N | NM_001128595 | **-1.64** | **1.51** | **-1.631** | **-2.291** |
| CD22 | NM_001185099 | -1.29 | 1.17 | -1.003 | **-2.294** |
| LOC105375172 | XR_927075 | -1.34 | **1.59** | **-1.824** | **-2.361** |
| PTGS2 | NM_000963 | 1.14 | **2.49** | 1.022 | **-2.390** |
| MUC13 | NM_033049 | **-1.83** | 1.20 | **-1.697** | **-2.415** |
| SPTLC3 | NM_018327 | -1.26 | **1.73** | -1.097 | **-2.470** |
| CP | NM_000096 | -1.41 | **1.53** | **-1.987** | **-2.485** |
| RNF43 | NM_001305544 | **-1.50** | **1.50** | **-1.525** | **-2.536** |
| CDH17 | NM_001144663 | **-1.91** | 1.01 | **-2.719** | **-2.627** |
| VTRNA2-1 | NR_030583 | **-2.15** | **-1.83** | **-2.717** | **-2.663** |
| ABLIM1 | XM_011539805 | **-2.12** | 1.09 | **-1.667** | **-2.674** |
| APOH | NM_000042 | -1.28 | 1.36 | **-1.770** | **-2.689** |
| AGR2 | NM_006408 | -1.44 | 1.34 | -1.414 | **-2.708** |
| TXNIP | NM_001313972 | -1.43 | -1.36 | **-2.626** | **-2.782** |
| MIR3189 | NR_036156 | 1.31 | **1.81** | -1.321 | **-2.815** |
| TM4SF20 | NM_024795 | **-1.95** | 1.03 | **-2.836** | **-2.845** |
| LOC101927630 | NR_110013 | **-1.81** | 1.35 | **-2.208** | **-2.943** |
| ANXA13 | NM_001003954 | **-1.99** | 1.15 | **-2.610** | **-3.591** |
| FGB | NM_001184741 | **-1.90** | **1.83** | **-2.665** | **-3.593** |
| FGA | NM_000508 | -1.33 | **2.48** | **-2.067** | **-4.120** |
| FGG | NM_000509 | **-1.65** | **2.37** | **-2.292** | **-4.879** |
|  |  |  |  |  |  |
