# Supplementary material for: Active PLK1-driven metastasis is amplified by TGF-β signaling that forms a positive feedback loop in non-small cell lung cancer
Source: Oncogene. 2019 Sep 23;39(4):767–85. doi: 10.1038/s41388-019-1023-z (PMC6976524; doi:10.1038/s41388-019-1023-z)
Supplement: Supplementary file 3 — Supplemental Table S2 [file 41388_2019_1023_MOESM3_ESM.doc]

**Supplementary Table S2. Downregulated genes targeted by MIR3167 in cells expressing wild type or active T210D PLK1.**

| Gene name | Gene Access No. | Noninvasive WT | Noninvasive TD | Invasive WT | Invasive TD |
| --- | --- | --- | --- | --- | --- |
| ST8SIA4 | NM_005668 | -1.25 | 1.24 | -1.386 | -2.131 |
| TNFRSF9 | NM_001561 | -1.36 | -1.54 | -1.441 | -1.668 |
| PMP22 | NM_000304 | -1.01 | 1.44 | 1.040 | -1.601 |
| DNAJC12 | NM_021800 | 1.00 | 1.65 | -1.059 | -1.581 |
| FOXA2 | NM_021784 | -1.06 | 1.03 | -1.072 | -1.386 |
| HKDC1 | NM_025130 | -1.08 | -1.19 | -1.123 | -1.326 |
| DENND1B | NM_001195215 | -1.41 | 1.10 | -1.037 | -1.237 |
| RNF113B | NM_178861 | 1.02 | 1.06 | 1.039 | -1.223 |
| WDR72 | NM_001277176 | -1.11 | -1.04 | -1.244 | -1.198 |
| NAP1L3 | NM_004538 | -1.24 | -1.00 | 1.137 | -1.165 |
| KRT20 | NM_019010 | -1.05 | 1.37 | 1.087 | -1.160 |
| KRTAP5-9 | NM_005553 | 1.01 | 1.09 | 1.136 | -1.136 |
| ZNF544 | ENST00000595981 | 1.00 | 1.17 | 1.334 | -1.124 |
| SLAMF6 | NM_001184714 | 1.01 | -1.01 | -1.143 | -1.122 |
| RBM12B-AS1 | NR_027259 | 1.04 | -1.01 | -1.292 | -1.121 |
| TRAPPC8 | NM_001003715 | -1.41 | -1.01 | 1.046 | -1.116 |
| ATP6V1G3 | NM_133262 | -1.06 | -1.06 | -1.139 | -1.106 |
| C1orf195 | NM_001278501 | -1.19 | -1.07 | -1.087 | -1.104 |
| VPS52 | NM_001289174 | -1.00 | -1.06 | -1.114 | -1.097 |
| STARD4 | NM_001308056 | -1.97 | -1.05 | -1.091 | -1.096 |
| PRAMEF20 | NR_039854 | -1.05 | -1.06 | -1.060 | -1.091 |
| EFHC2 | NM_025184 | 1.10 | 1.14 | 1.080 | -1.088 |
| ZNF322 | NM_001242797 | -1.36 | -1.05 | 1.023 | -1.087 |
| ZNF586 | NM_001077426 | -1.02 | 1.03 | 1.001 | -1.075 |
| GLOD4 | NM_016080 | 1.00 | 1.05 | 1.075 | -1.071 |
| EIF2D | NM_001201478 | -1.12 | -1.10 | 1.012 | -1.069 |
| HUWE1 | NM_031407 | -1.03 | 1.06 | -1.018 | -1.066 |
| TMEM230 | NM_001009923 | 1.02 | 1.12 | 1.116 | -1.065 |
| NUDT4 | NM_001301022 | -1.29 | -1.01 | 1.151 | -1.064 |
| SYT4 | NM_020783 | 1.10 | 1.10 | -1.265 | -1.063 |
| SERHL2 | NM_001284334 | 1.02 | -1.19 | -1.111 | -1.057 |
| PJA1 | NM_001032396 | -1.13 | -1.15 | 1.007 | -1.054 |
| FREM3 | NM_001168235 | -1.04 | -1.08 | -1.185 | -1.051 |
| ZNF786 | NM_152411 | -1.14 | -1.02 | 1.046 | -1.039 |
| STYK1 | NM_018423 | -1.12 | -1.02 | -1.083 | -1.033 |
| WDR77 | NM_024102 | -1.02 | 1.04 | 1.117 | -1.010 |
| S100Z | NM_130772 | 1.09 | 1.25 | 1.223 | -1.007 |
