# Supplementary material for: Active PLK1-driven metastasis is amplified by TGF-β signaling that forms a positive feedback loop in non-small cell lung cancer
Source: Oncogene. 2019 Sep 23;39(4):767–85. doi: 10.1038/s41388-019-1023-z (PMC6976524; doi:10.1038/s41388-019-1023-z)
Supplement: Supplementary file 4 — Supplemental Table S3 [file 41388_2019_1023_MOESM4_ESM.doc]

**Supplementary Table S3.** Sequences of forward (F) and reverse (R) primers used for RT-PCR amplification.

| Target Gene | Primer | Sequences |
| --- | --- | --- |
| *PLK1* | Forward  Reverse | 5’- AAGAGATCCCGGAGGTCCTA -3’  5’- TCATTCAGGAAAAGGTTGCC -3’ |
| *CDH1* | Forward  Reverse | 5’- ACCACCTCCACAGCCACC -3’  5’- GTCCAGTTGGCACTCGCC -3’ |
| *CDH2* | Forward  Reverse | 5’- ACAGTGGCCACCTACAAAGG -3’  5’- CCGAGATGGGGTTGATAATG -3’ |
| *VIM* | Forward  Reverse | 5’- GAGAACTTTGCCGTTGAAGC -3’  5’- GCTTCCTGTAGGTGGCAATC -3’ |
| *SNAI1* | Forward  Reverse | 5’- GGAAGCCTAACTACAGCGAG -3’  5’- CAGAGTCCCAGATGAGCATTG -3’ |
| *SNAI2* | Forward  Reverse | 5’- ACGCCCAGCTACCCAATG -3’  5’- AGGGCGCCCAGGCTCACATA -3’ |
| *ZEB1* | Forward  Reverse | 5’- TGGGATCAACCACCAATGG -3’  5’- AAGTAACCCTGTGTATTTCTGGATGA -3’ |
| *TWIST* | Forward  Reverse | 5’- GGACAAGCTGAGCAAGATTCAGA -3’  5’- TCTGGAGGACCTGGTAGAGGAA -3’ |
| *LCE3D* | Forward  Reverse | 5’- CAAGTGTCCCTCACCCAAGT -3’  5’- GTCACAGGAGTTGGGCCTCT -3’ |
| *TNFAIP6* | Forward  Reverse | 5’- TGCTGCTGGATGGATGGCTA -3’  5’- CACTCCTTTGCGTGTGGGTT -3’ |
| *LAMC2* | Forward  Reverse | 5’- GCCTTTTGGCACCTGTATTC -3’  5’- CAGGATTCTCATCCCCTGAA -3’ |
| *CD44* | Forward  Reverse | 5’- TGC CGC TTT GCA GGT GTA TT-3’  5’- CCG ATG CTC AGA GCT TTC TCC-3’ |
| *GAPDH* | Forward  Reverse | 5’- TAAAGGGCATCCTGGGCTACACT -3’  5’- TTACTCCTTGGAGGCCATGTAGG -3’ |
