# Supplementary figures and images for: Active PLK1-driven metastasis is amplified by TGF-β signaling that forms a positive feedback loop in non-small cell lung cancer
Source: Oncogene. 2019 Sep 23;39(4):767–85. doi: 10.1038/s41388-019-1023-z (PMC6976524; doi:10.1038/s41388-019-1023-z)

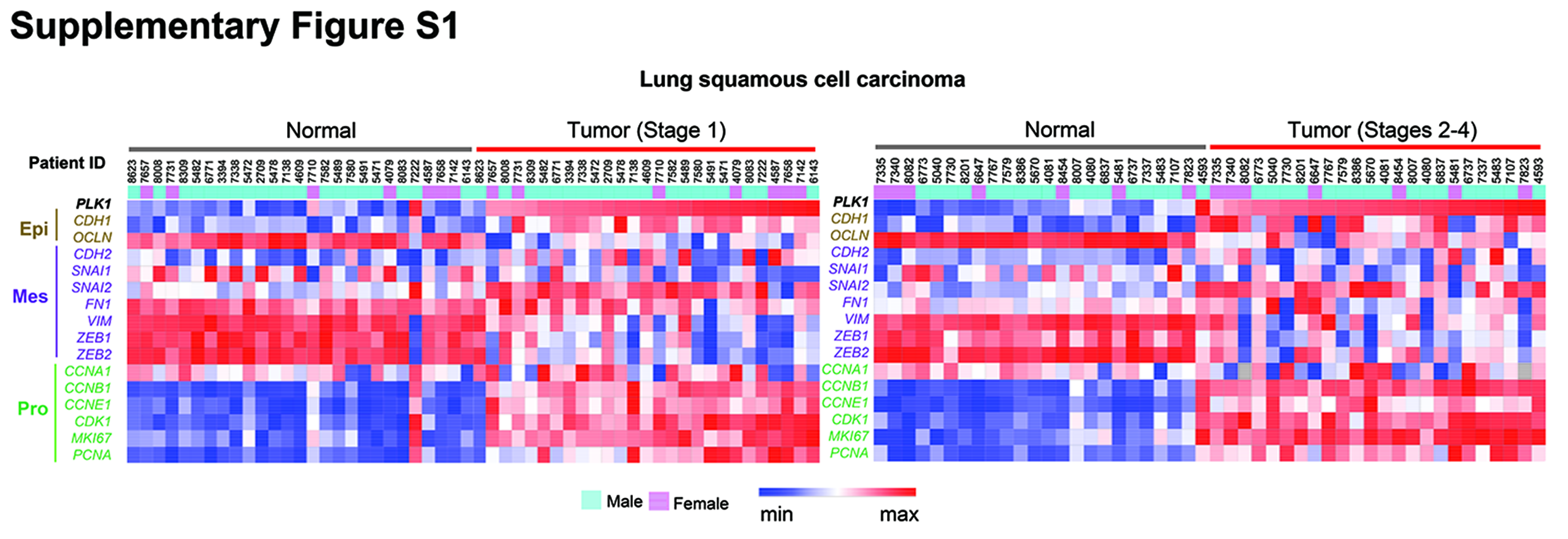

Supplement: Supplementary file 5 — Supplementary Figure S1 [file 41388_2019_1023_MOESM5_ESM.tif]

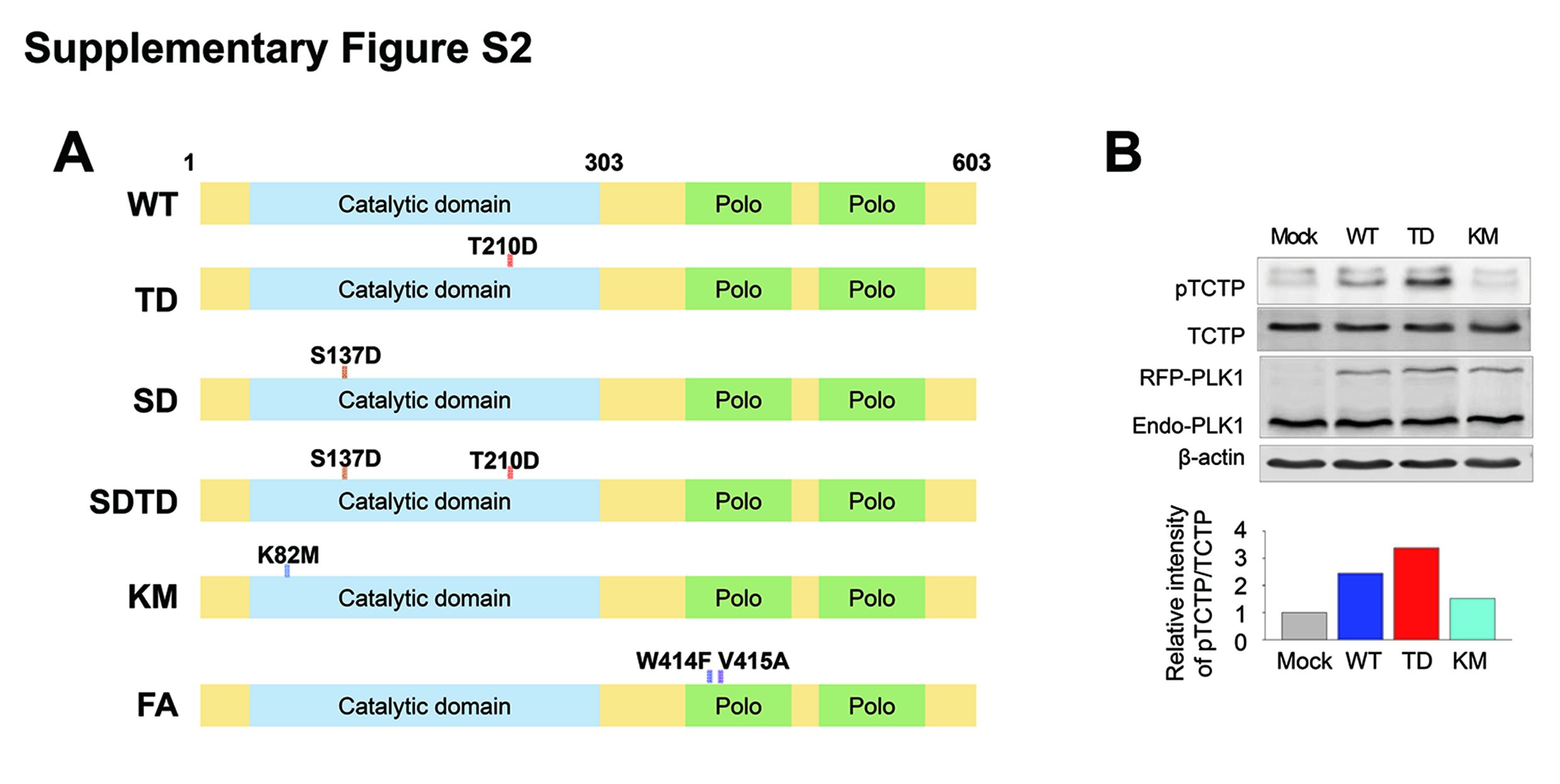

Supplement: Supplementary file 6 — Supplementary Figure S2 [file 41388_2019_1023_MOESM6_ESM.tif]

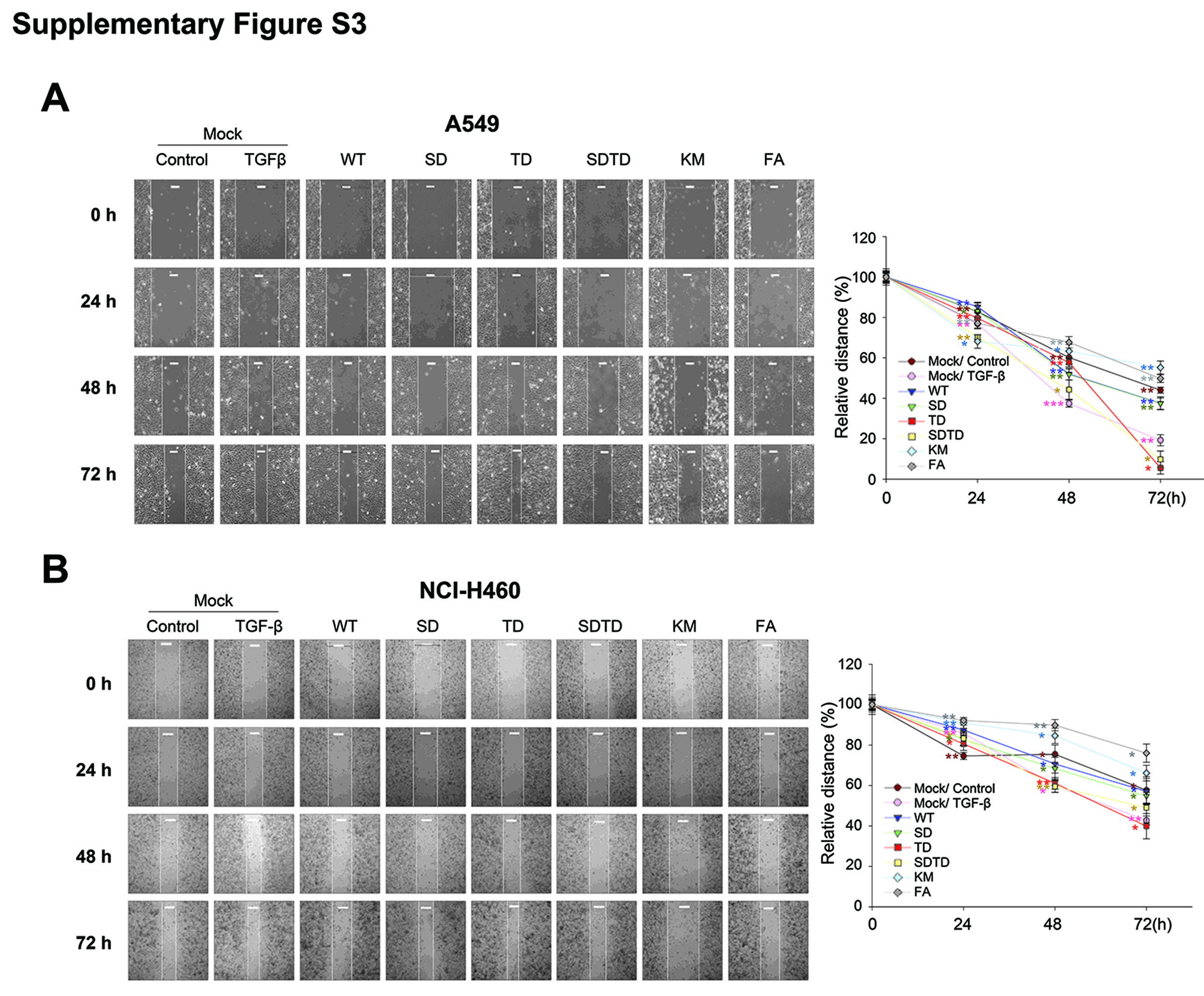

Supplement: Supplementary file 7 — Supplementary Figure S3 [file 41388_2019_1023_MOESM7_ESM.tif]

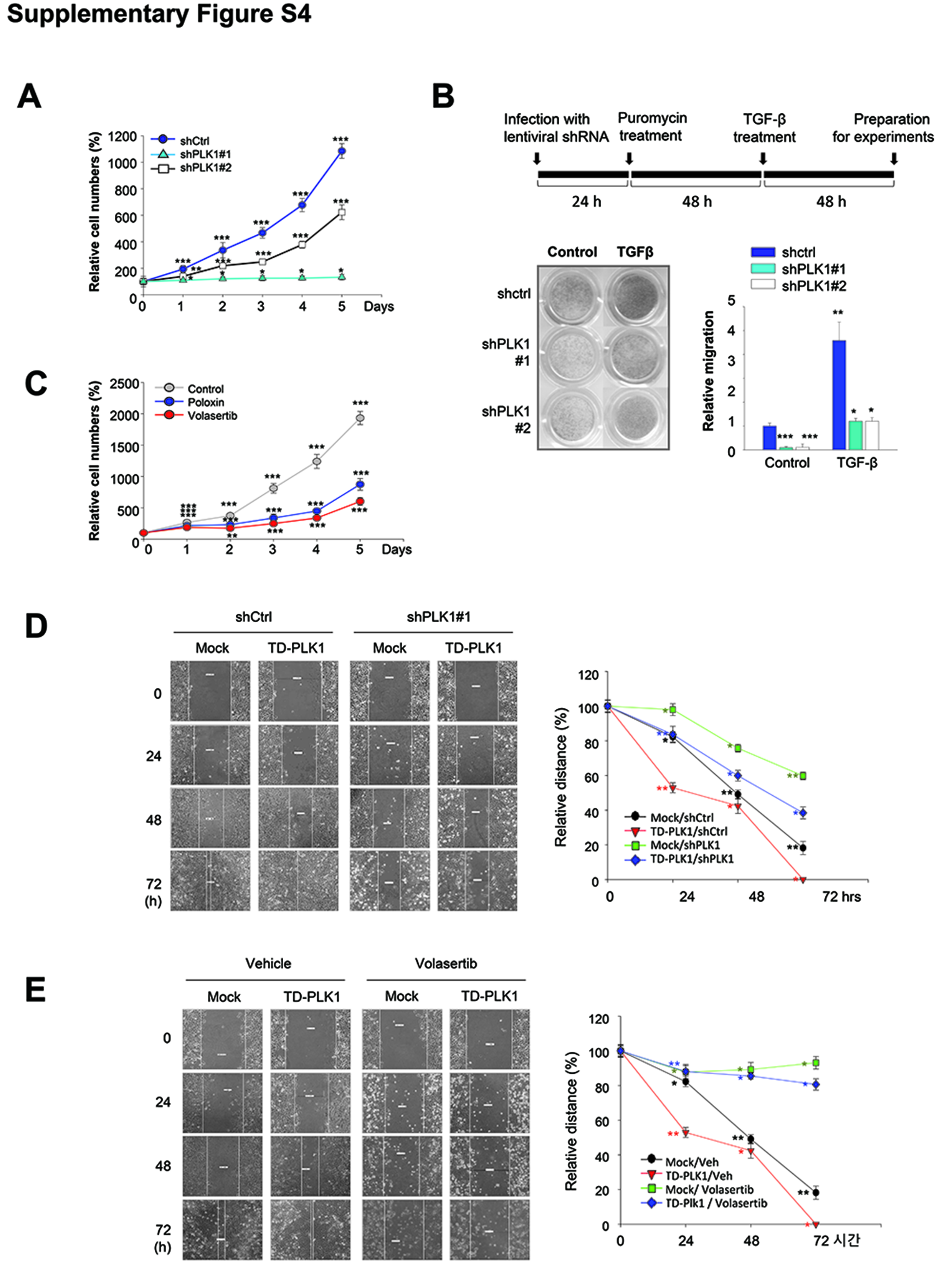

Supplement: Supplementary file 8 — Supplementary Figure S4 [file 41388_2019_1023_MOESM8_ESM.tif]

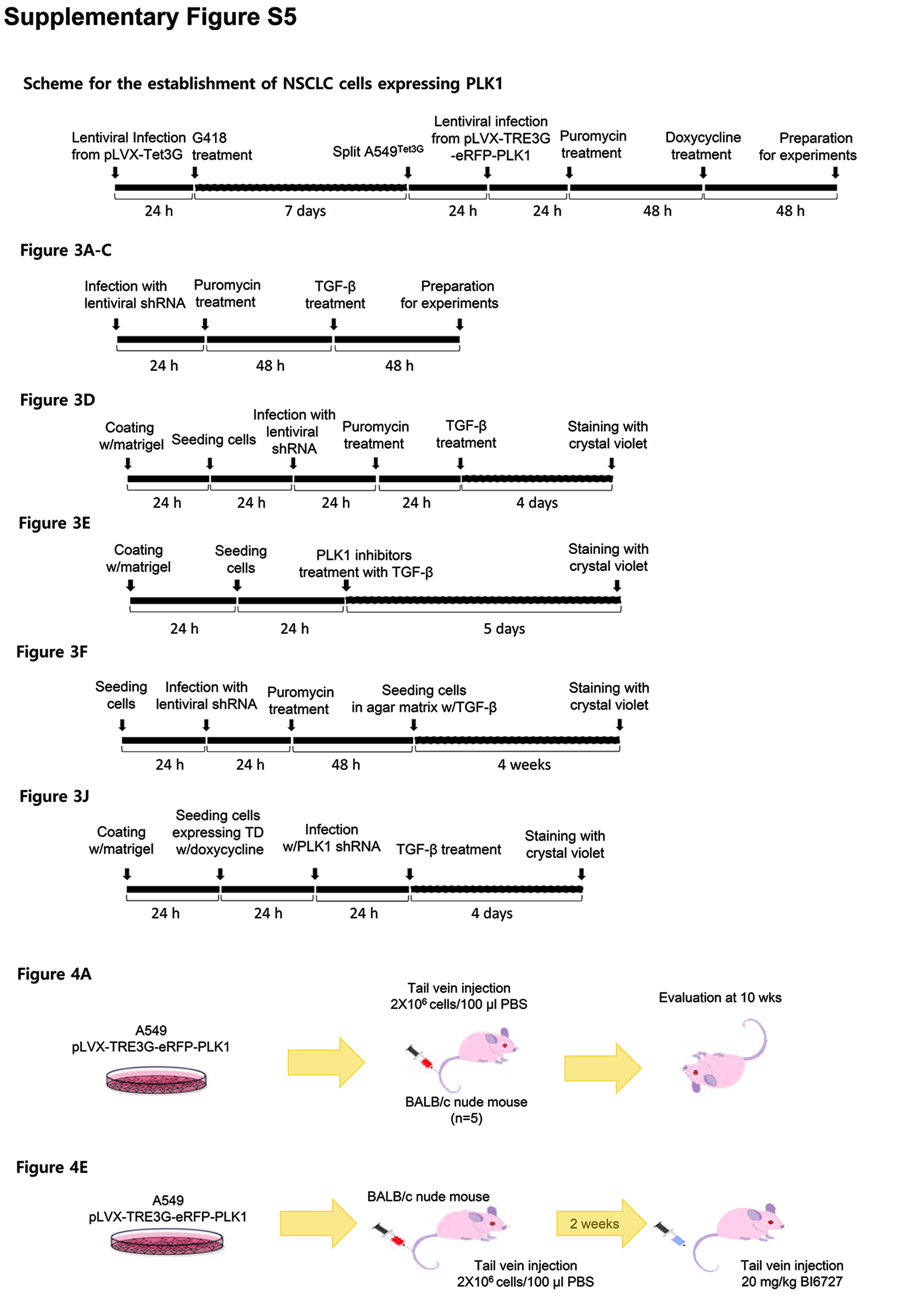

Supplement: Supplementary file 9 — Supplementary Figure S5 [file 41388_2019_1023_MOESM9_ESM.tif]

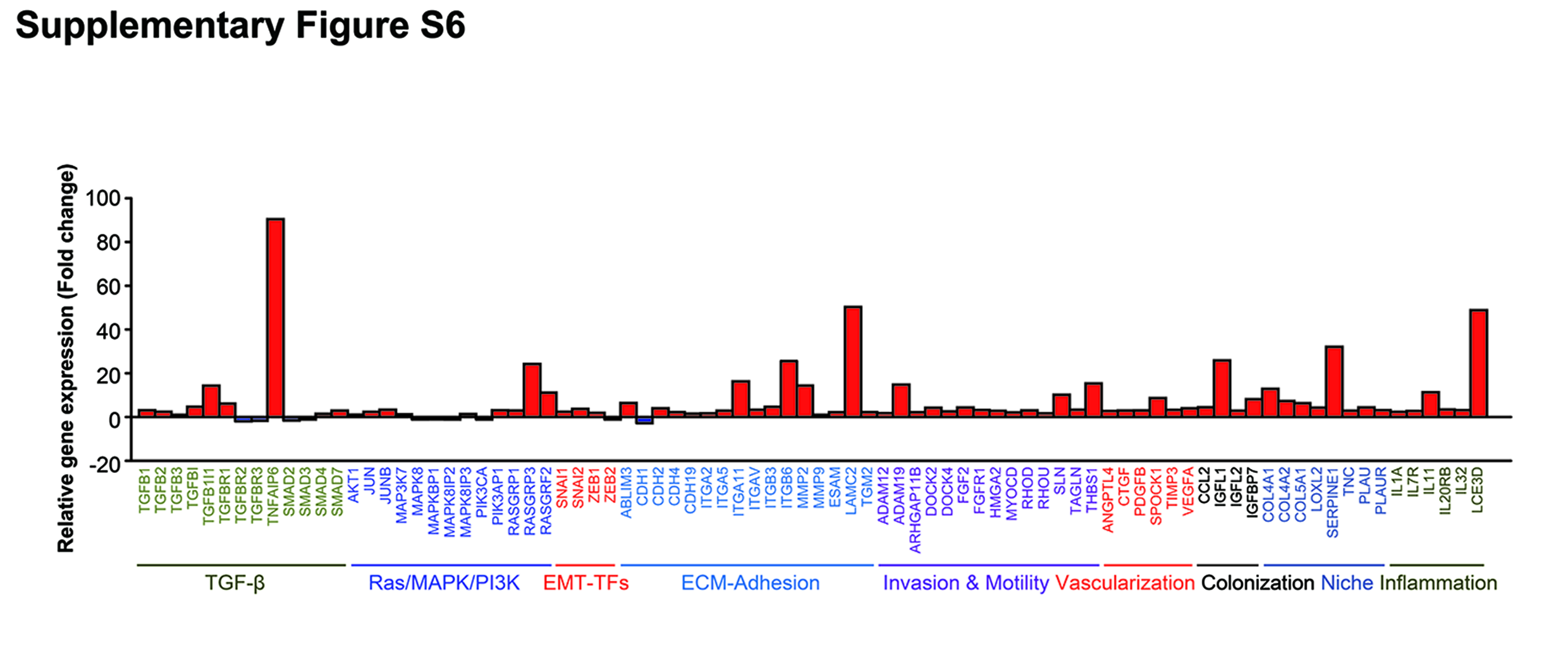

Supplement: Supplementary file 10 — Supplementary Figure S6 [file 41388_2019_1023_MOESM10_ESM.tif]

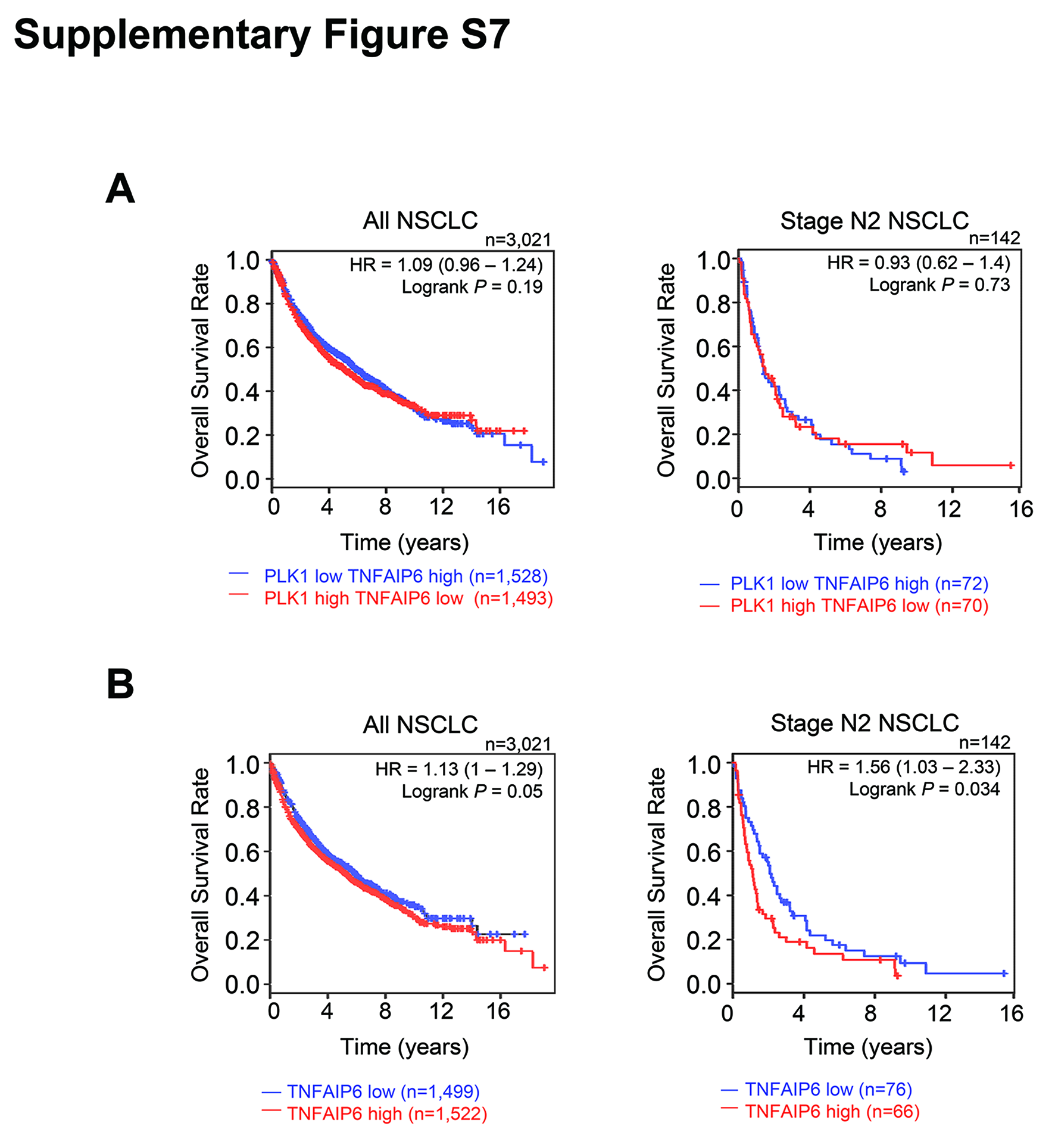

Supplement: Supplementary file 11 — Supplementary Figure S7 [file 41388_2019_1023_MOESM11_ESM.tif]

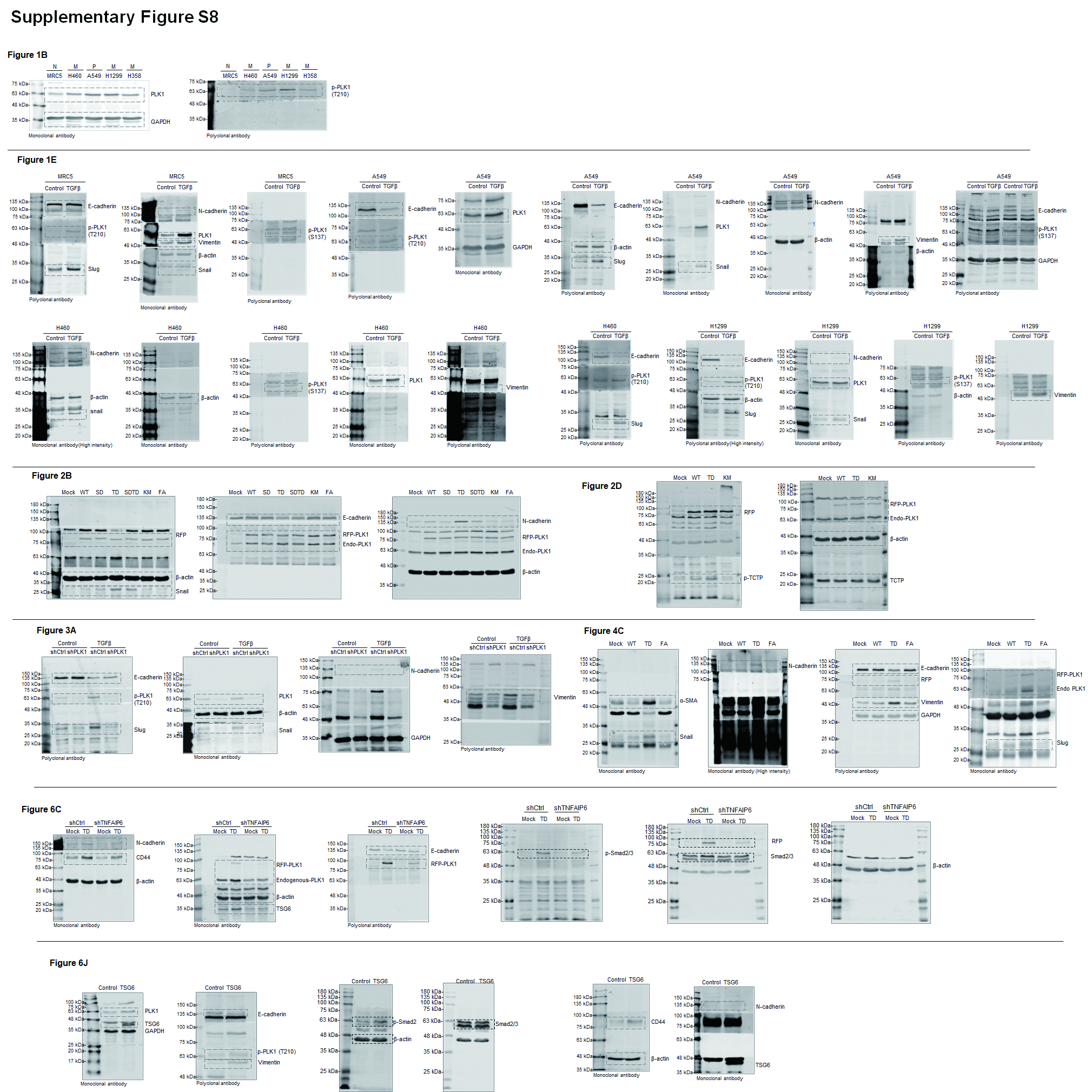

Supplement: Supplementary file 12 — Supplementary Figure S8 [file 41388_2019_1023_MOESM12_ESM.tif]
